# SOX4 and SMARCA4 cooperatively regulate PI3k signaling through transcriptional activation of TGFBR2

**DOI:** 10.1038/s41523-021-00248-2

**Published:** 2021-04-09

**Authors:** Gaurav A. Mehta, Steven P. Angus, Christen A. Khella, Kevin Tong, Pooja Khanna, Shelley A. H. Dixon, Michael P. Verzi, Gary L. Johnson, Michael L. Gatza

**Affiliations:** 1 Department of Radiation Oncology, Robert Wood Johnson Medical School, New Brunswick, NJ USA; 2 Rutgers Cancer Institute of New Jersey, New Brunswick, NJ USA; 3 Department of Pediatrics, Indiana University School of Medicine, Indianapolis, IN USA; 4 Department of Genetics, Human Genetics Institute of New Jersey, Rutgers, The State University of New Jersey, Piscataway, NJ USA; 5 Department of Pharmacology, University of North Carolina School of Medicine, Chapel Hill, NC USA

**Keywords:** Breast cancer, Breast cancer

## Abstract

Dysregulation of PI3K/Akt signaling is a dominant feature in basal-like or triple-negative breast cancers (TNBC). However, the mechanisms regulating this pathway are largely unknown in this subset of aggressive tumors. Here we demonstrate that the transcription factor SOX4 is a key regulator of PI3K signaling in TNBC. Genomic and proteomic analyses coupled with mechanistic studies identified *TGFBR2* as a direct transcriptional target of SOX4 and demonstrated that TGFBR2 is required to mediate SOX4-dependent PI3K signaling. We further report that SOX4 and the SWI/SNF ATPase SMARCA4, which are uniformly overexpressed in basal-like tumors, form a previously unreported complex that is required to maintain an open chromatin conformation at the *TGFBR2* regulatory regions in order to mediate *TGFBR2* expression and PI3K signaling. Collectively, our findings delineate the mechanism by which SOX4 and SMARCA4 cooperatively regulate PI3K/Akt signaling and suggest that this complex may play an essential role in TNBC genesis and/or progression.

## Introduction

Triple-negative breast cancer (TNBC), which is largely synonymous with the basal-like molecular subtype of breast cancer, accounts for 10–15% of all breast-cancer cases^1^. These aggressive tumors are predominant in younger women and women of African American descent and are characterized by poor clinical outcome, accounting for ~1-in-4 breast-cancer-related deaths^2,3^. Previous studies, including those from The Cancer Genome Atlas (TCGA) project and our own work, have reported increased and uniform activation of phosphatidylinositol-3-OH kinase (PI3K) signaling in basal-like breast tumors^3–6^. This pathway mediates multiple oncogenic processes including proliferation, metabolism, motility, and genome instability^3,7,8^. While *PIK3CA*, which encodes for the oncogenic p110α catalytic subunit of the kinase, is the most commonly mutated gene in breast cancer^3^, activating mutations occur at a low incidence (~9%) in basal-like tumors suggesting that other mechanisms contribute to altered signaling in these tumors^3,9^. Consistent with this argument, multi-platform genomic analyses have identified copy-number alterations or mutations in repressors of PI3K signaling including *PTEN* (35%) as well as mutations in known drivers of the pathway including *EGFR* (7%), *ERBB2* (4%), *IGFR1* (2%), and others in basal-like tumors^3,6^. Despite these observations, predominant mechanisms regulating activation of this pathway have not been identified in TNBC or basal-like tumors. While inhibition of PI3K/Akt/MTOR signaling has been shown to be effective in preclinical studies and in ER+ breast cancers, similar clinical success has not been achieved for TNBC^7,10–15^. These clinical results suggest that understanding and targeting these additional mechanisms of PI3K pathway regulation and/or complementary pathways will be important for optimizing therapeutic strategies for TNBC patients.

SOX4 is a well-established oncogene and a member of the SOX C family of SRY-related HMG-box (SOX) transcription factors^16^. Increased expression of *SOX4* has been shown to be associated with malignant transformation and metastasis in several cancer types including breast^5,17–20^, prostate^21,22^, acute lymphoblastic leukemia^23^, and melanoma^24^. In the context of breast cancer, overexpression of SOX4 corresponds with poor overall survival, particularly in basal-like or TNBC tumors^18,20,25^. The pro-oncogenic function of SOX4 in breast cancer is widely attributed to its ability to modulate epithelial-to-mesenchymal transition (EMT), activation of multiple pro-proliferative or pro-survival signaling pathways, increased angiogenesis as well as its role in regulating cancer cell stemness^16–20^. Consistent with the observed effect of SOX4 on multiple oncogenic signaling pathways, we recently showed that SOX4 is an essential regulator of PI3K/Akt signaling in basal-like tumors^5^. These previous studies demonstrated that *SOX4* expression is increased in basal-like breast tumors with high PI3K activity, independent of genomic alteration in commonly altered PI3K/Akt regulatory genes including PIK3CA and PTEN, and that siRNA-mediated silencing of SOX4 abrogates the activation of this pathway in basal-like cell lines; however, the mechanism(s) by which SOX4 regulates this pathway in TNBC remains unknown.

SWI*/*SNF chromatin remodeling enzymes are multi-subunit and evolutionarily conserved complexes that remodel chromatin by utilizing energy derived from ATP hydrolysis and render sites accessible to transcription factor binding in order to promote gene expression^26,27^. Mutations in subunits of the SWI/SNF chromatin remodeling complexes have been reported in nearly 20% of all human cancers, highlighting their important roles in tumorigenesis^28–30^. Consistent with these general observations, recent studies have demonstrated that increased expression of SMARCA4, which encodes the catalytic ATPase subunit of the SWI/SNF complex, is associated with poor prognosis in breast cancer^31–33^. Mechanistically, SMARCA4 has been reported to play an oncogenic role in breast cancer by regulating critical aspects of breast-cancer biology including lipid metabolism, proliferation, and resistance to chemotherapeutic drugs^34,35^. Although SMARCA4 has been shown to regulate several oncogenic properties in breast cancer, the mechanisms by which it promotes these processes in mammary tumorigenesis, including potential effects on PI3K/Akt signaling, remains unknown.

The goal of the current study was to define the mechanism and co-factors required for SOX4-mediated activation of PI3K/Akt signaling in TNBC. To achieve this goal, mRNA sequencing (RNAseq) and kinome profiling coupled with molecular analyses identified and determined that SOX4-mediated activation of PI3K/Akt signaling is dependent on transcriptional regulation of transforming growth factor β receptor 2 (*TGFBR2*) by SOX4. At the molecular level, our data demonstrate that SOX4 recruits SMARCA4 to the *TGFBR2* promoter and enhancer in order to mediate chromatin remodeling and activation of *TGFBR2* expression. Given that our data indicate that SOX4 and SMARCA4 are uniformly activated in basal-like breast tumors, our findings not only delineate a mechanism by which SOX4 and SMARCA4 mediate PI3K activity in TNBC or basal-like breast cancers but have also identified a novel complex by which SOX4 cooperates with SMARCA4 to modulate transcriptional activation and oncogenic signaling in basal-like breast cancer.

## Results

### SOX4 expression is upregulated in basal-like tumors and associated with increased PI3K signaling

**Fig. 1:**
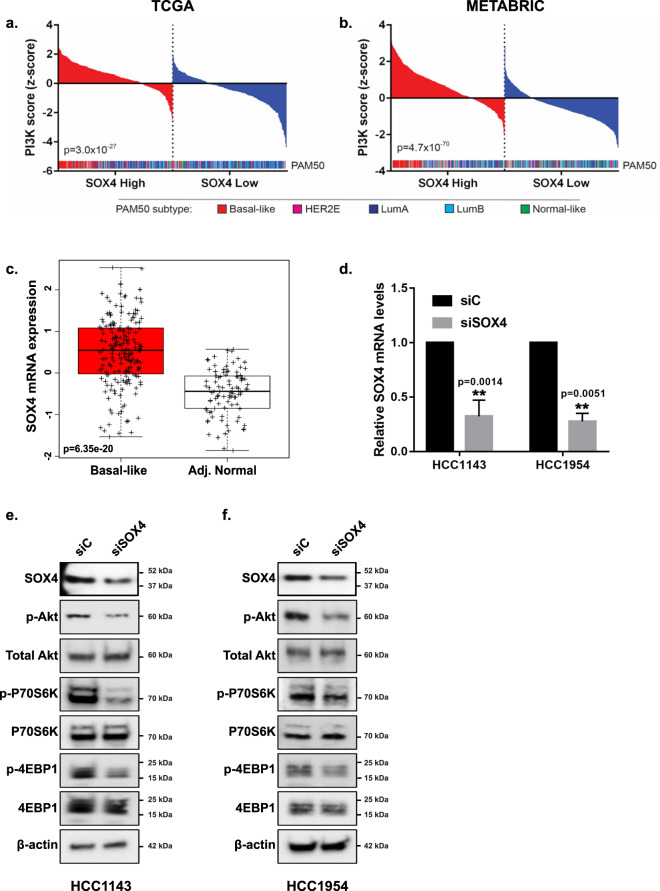
SOX4 regulates the PI3K/Akt pathway activity in basal-like breast cancer. PI3K pathway activity is significantly upregulated in tumor samples with high *SOX4* expression (top quartile) compared to samples with low (bottom quartile) *SOX4* expression in the **a** TCGA (*n* = 1031, *p* = 3.0 × 10^−27^) and **b** METABRIC (*n* = 1991, *p* = 4.7 × 10^−70^) datasets. PAM50 subtype is shown in the inset of **a** and **b** and demonstrates that samples with high *SOX4* expression are predominantly basal-like tumors (red) in both datasets. **c** SOX4 expression is significantly upregulated in 88.1% of in basal-like breast tumors (*n* = 179) compared to the median expression in adjacent normal tissue (*n* = 94) from the TCGA dataset (*p* = 6.35 × 10^−^^20^). **d** siRNA-mediated silencing of SOX4 significantly reduced SOX4 expression in HCC1143 (*p* = 0.001) and HCC1954 (*p* = 0.005) TNBC cell lines. Representative western blot analysis demonstrating significant reduction in SOX4, pAkt, pS6K, and p4EBP1 expression in both **e** HCC1143 and **f** HCC1954 cell lines.

Previous studies have demonstrated that basal-like tumors are characterized by high PI3K/Akt signaling^3–5^. More recently, we reported that SOX4 can mediate PI3K and Akt signaling in TNBC cell lines^5^. In order to demonstrate the relationship between SOX4 expression and aberrant PI3K/Akt signaling, as well as the association between SOX4 expression and molecular subtype, we examined these relationships in human breast tumors. Patient tumor samples from TCGA (*n* = 1031)^3^ and Molecular Taxonomy of Breast Cancer International Consortium (METABRIC, *n* = 1992)^36^ cohorts were scored for PAM50 subtype^37^, and PI3K pathway activity was calculated using a previously published PI3K gene expression signature (Supplementary Data 1 and 2)^38,39^. As illustrated in Fig. 1, when tumor samples were dichotomized into *SOX4* high (top quartile) and low (bottom quartile) expressing subgroups, PI3K signaling was significantly upregulated in both the TCGA (Fig. 1a; *p* = 3.0 × 10^−27^) and METABRIC (Fig. 1b; *p* = 4.7 × 10^−70^) cohorts in SOX4 high expressing tumors. As expected^5^, increased SOX4 expression was predominantly observed in basal-like tumors (Fig. 1a, b) and analysis of the TCGA dataset demonstrated that more than 88.1% of basal-like tumors (*n* = 179) were characterized by increased (*p* = 6.35 × e^−20^) SOX4 expression relative to the median expression in adjacent normal breast tissue (*n* = 94) (Fig. 1c and Supplementary Data 1).

In order to identify cell lines for in vitro studies, we used data from publicly available gene expression and proteome profiling studies to examine *SOX4* mRNA (Supplementary Fig. 1a) and protein (Supplementary Fig. 1b) expression in a panel of breast-cancer cell lines. Western blot analyses were performed to validate SOX4 expression in a subset of basal-like cell lines that demonstrated relatively high (HCC1143, HCC1954, MDAMB468, HCC1395, HCC38, HCC70) or low (BT20, MDAMB231) SOX4 expression (Supplementary Fig. 1c). Based on these analyses, as well as previously published PI3K activity scores, HCC1143 and HCC1954 were identified as basal-like breast-cancer cell lines with high SOX4 expression and high PI3K activity and selected for in vitro studies^5,40^. As expected RNAi-mediated silencing reduced SOX4 mRNA expression by 67.3% in HCC1143 (*p* = 0.0014) and 72.3% in HCC1954 (*p* = 0.0051) cells. This corresponded with a significant 34.6% (*p* = 0.0019) and 40.1% (*p* = 0.0067) reduction in protein expression in each cell line (Fig. 1e, f and Supplementary Fig. 2a, b). Consistent with predictions from in silico analyses of human tumors, siRNA-mediated silencing of SOX4 in HCC1143 (*p* = 0.0019) cells significantly reduced Akt phosphorylation at Ser473 (*p* = 0.0002) and decreased phosphorylation of downstream PI3K pathway proteins including pP70S6K at Thr389 (*p* = 0.0006) and p4EBP1 at Thr37/46 (*p* = 0.0043); siRNA-mediated silencing of SOX4 did not have an effect on total Akt, P70S6K, or 4EBP1 levels (Fig. 1e and Supplementary Fig. 2a). Likewise, siRNA-mediated silencing of SOX4 in HCC1954 cells (*p* = 0.0067) resulted in a similar reduction in Akt signaling as evident by decreased phosphorylation of pAkt (Ser473; *p* = 0.0181), pP70S6K (Thr389; *p* = 0.0222), and p4EBP1 (Thr37/46; *p* = 0.0108) with no concomitant change in total Akt, P70S6K, or 4EBP1 expression (Fig. 1e and Supplementary Fig. 2b). In order to validate the specificity of these findings, we engineered HCC1143 and HCC1954 cells to express one of two unique tetracycline-inducible shRNA against SOX4 (referred to as sh-1 and sh-2); as expected, parental and tet-inducible shRNA expressing HCC1143 and HCC1954 cells show similar basal levels of SOX4, pAkt, and total Akt protein expression (Supplementary Fig. 2c, d). As illustrated in Supplementary Fig. 2e, doxycycline (dox) treatment resulted in significant reduction of *SOX4* mRNA levels in sh-1 (83.0% reduction, *p* = 0.0049) or sh-2 (65.01% reduction, *p* = 0.0099) expressing HCC1143 cells and sh-1 (86.45% reduction, *p* = 0.0008) or sh-2 (73.0% reduction, *p* = 0.0013) expressing HCC1954 cell lines (Supplementary Fig. 2e). In agreement with siRNA knockdown, shRNA-mediated depletion of SOX4 resulted in the significant decrease in SOX4 protein expression as well as Akt (S473) phosphorylation in both HCC1143 (Supplementary Fig. 2f) and HCC1954 (Supplementary Fig. 2g) cell lines.

### Identification of the SOX4-activated kinome

**Fig. 2:**
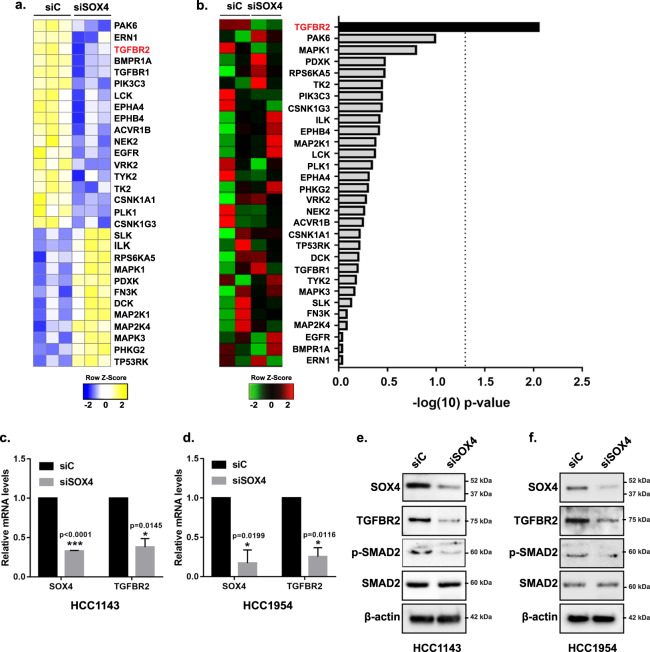
TGFBR2 is a downstream target gene of SOX4. **a** Profiling of the drug-able kinome by MIB/MS analysis in HCC1143 cells identified 18 kinases with significantly reduced expression and/or affinity for Type I kinase inhibitor bait (*p* < 0.05) and 12 kinases with increased expression/affinity following siRNA-mediated silencing of SOX4 (*n* = 3, 96 h) relative to siControl-treated samples (*n* = 3); kinase enrichment score is shown in yellow and kinase depletion score in blue. **b** Differential gene expression analyses of RNAseq data for the significantly altered kinases from MIB/MS analysis from siSOX4- and siControl-treated HCC1143 cells (96 h); high mRNA expression is shown in red and low expression in green. **c** HCC1143 and **d** HCC1954 cells demonstrate significant reduction (unpaired t-test) in SOX4 (*p* < 0.0001; *p* = 0.019) and TGFBR2 (*p* = 0.014; *p* = 0.012) expression in siSOX4-treated cells compared to siControl-treated cell by qRT-PCR; data are presented as mean ± SD and normalized for GAPDH (*n* = 3). **e** HCC1143 and **f** HCC1954 cells showing reduced SOX4 and TGFBR2 protein expression by western blot analyses in siSOX4-treated cells relative to siControl-treated cells.

Given that SOX4 is a transcription factor, we hypothesized that SOX4 mediates its effects on PI3K/Akt signaling by regulating expression of upstream kinases. In order to identify kinases regulated by SOX4 that contribute to increased PI3K/Akt signaling, we performed kinome profiling using multiplexed inhibitor beads coupled with mass spectrometry (MIB/MS). MIB/MS is a chemical-proteomic-based approach, which utilizes multiple kinase inhibitors to enrich the functional kinome from tumor or cell lysates by affinity chromatography followed by quantitative mass spectrometry (MS)^41^. Using this approach, we identified 208 enriched kinases in siSOX4 or siControl-treated HCC1143 cells. Of these, the MIB binding of 30 kinases was significantly (*p* < 0.05) altered following siRNA-mediated silencing of *SOX4* relative to siControl-treated cells. Eighteen kinases, including BMPR1A^42^, LCK^43^, ACVR1B^44^, and EGFR^45^, which are known regulators of PI3K signaling, were significantly depleted while twelve kinases including several belonging to MAPK family showed increased enrichment following siSOX4 treatment (Fig. 2a and Supplementary Data 3).

To determine if any of the depleted kinases from MIB/MS analysis are transcriptional targets of SOX4, and thus directly regulated by SOX4 activity, we performed transcriptome profiling by RNA sequencing in HCC1143 cells treated with siRNA against SOX4 or a control siRNA. Differential gene expression analyses of RNAseq data identified TGFBR2 (*p* = 0.009) as the only gene among the MIB/MS hits that was significantly downregulated following siRNA-mediated silencing of *SOX4* (Fig. 2b and Supplementary Data 4).

In order to validate these omics-based analyses, the effect of siRNA-mediated knockdown of SOX4 on TGFBR2 expression was examined in HCC1143 and HCC1954 cell lines. Quantitative real-time PCR (qRT-PCR) analysis confirmed downregulation of *TGFBR2* expression at the transcript level in both HCC1143 (62.0% reduction, *p* = 0.014) (Fig. 2c) and HCC1954 (74.6% reduction, *p* = 0.011) (Fig. 2d); a similar percent reduction was also observed in these cells at the protein level in HCC1143 (Fig. 2e and Supplementary Fig. 3a; *p* = 0.005) and HCC1954 (Fig. 2f and Supplementary Fig. 3b; *p* = 0.007) cells. Importantly a significant reduction in p-SMAD2 was observed in both HCC1143 (Fig. 2e and Supplementary Fig. 3a; *p* = 0.01) and HCC1954 (Fig. 2f and Supplementary Fig. 3b; *p* = 0.01) cells indicating that TGFβ signaling is functionally inhibited by SOX4 repression. Consistent with these results, shRNA-mediated silencing of SOX4, using two independent tet-inducible shRNA, also resulted in a significant downregulation of TGFBR2 protein expression in both cell lines (Supplementary Fig. 3c and Supplementary Fig. 3d).

### SOX4-induced TGFBR2 expression promotes PI3K/Akt signaling

**Fig. 3:**
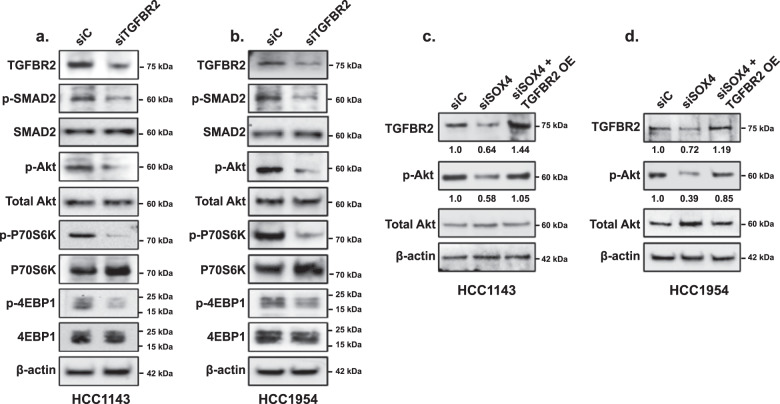
SOX4 mediates activation of the PI3K pathway through TGFBR2. **a** Western blot analysis showing downregulation of the PI3K pathway activity as indicated by decreased expression of PI3K pathway downstream components in HCC1143 following siTGFBR2 treatment (96 h) relative to siControl treatment. p-SMAD2 downregulation is used as a positive control for TGFBR2 protein depletion. **b** Similar results were observed in HCC1954 cells. **c** Lentiviral overexpression of TGFBR2 restores pAkt levels in siSOX4 (96 h) treated HCC1143 cells. **d** Consistent results were observed in HCC1954 cells.

To investigate the impact of TGFBR2 on SOX4 activation of the PI3K/Akt pathway in TNBC, we first inhibited TGFBR2 expression by siRNA independent of SOX4 perturbation. RNAi-mediated suppression of TGFBR2 significantly reduced PI3K signaling as apparent by decreased phosphorylation of downstream markers of the PI3K pathway including pAkt (Ser473) (HCC1143, *p* = 0.018; HCC1954, *p* = 0.007), pP70S6K (Thr389) (HCC1143, *p* = 0.023; HCC1954, *p* = 0.012), and p4EBP1 (Thr37/46) (HCC1143, *p* = 0.035; HCC1954, *p* = 0.025) in HCC1143 (Fig. 3a and Supplementary Fig. 4a) and HCC1954 (Fig. 3b and Supplementary Fig. 4b) cells. As shown in Fig. 3, total protein levels for Akt, P70S6K, and 4EBP1 remain unchanged and this effect was consistent in both cell lines (Fig. 3a, b).

In order to test the hypothesis that SOX4-mediated activation of PI3K signaling is dependent on TGFBR2 expression, SOX4 was depleted in HCC1143 or HCC1954 cell lines by siRNA while TGFBR2 was concurrently overexpressed by lentiviral transduction. As expected, SOX4 knockdown by RNAi (siSOX4) significantly reduced the expression of TGFBR2 and phosphorylation of Akt (Ser473) in both HCC1143 (Fig. 3c) and HCC1954 (Fig. 3d) cell lines. Importantly, we determined that TGFBR2 overexpression in conjunction with SOX4 knockdown was able to rescue PI3K/Akt signaling, as illustrated by restored pAkt (Ser473) levels, in each cell line (Fig. 3c, d).

### SOX4 regulates TGFBR2 expression by binding to the HMG-box domain in its promoter and enhancer regions

**Fig. 4:**
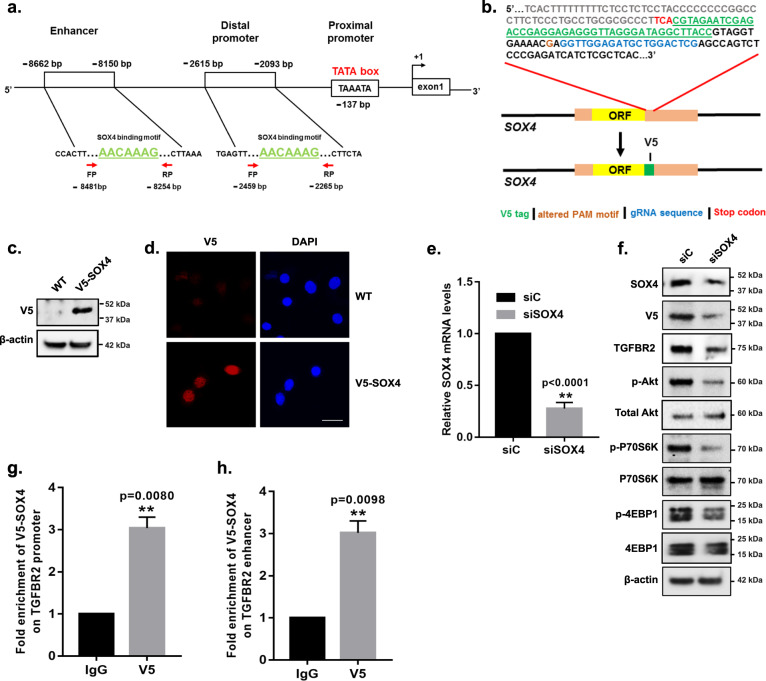
SOX4 binds to regulatory sequences in *TGFBR2* promoter and enhancer region. **a** Schematic representation of the *TGFBR2* regulatory regions upstream from the transcription start site. The proximal promoter with TATA box (red), the SOX4 binding motif, (AACAAAG) in the distal promoter and enhancer (green), and forward/reverse primer locations (red arrows) are noted. **b** Schematic of the CRISPR/Cas9 genome editing strategy used to insert a C-terminal V5 epitope tag (green) at the 3′ locus immediately upstream of the stop codon (red); the gRNA sequence (blue) and the altered PAM motif (brown) are highlighted. **c** Western blot and **d** immunofluorescence demonstrate V5 expression in the HCC1143 ^SOX4-V5^ cell line; no expression is observed in the parental cell. The scale bar is 100 µm. **e** qRT-PCR demonstrates that *SOX4* mRNA levels are significantly reduced by siSOX4 in HCC1143^SOX4-V5^ (*p* < 0.0001) relative to siControl transfected cells (50 nm, 96 h). **f** Western blot analyses demonstrated reduced protein expression of SOX4, V5, TGFBR2, and phosphorylated PI3K pathway marker proteins in HCC1143 ^SOX4-V5^ cells transfected with siSOX4 relative to siControl-treated cells (96 h). **g** ChIP-qPCR using anti-V5 antibody demonstrated a 3.03-fold enrichment (*p* = 0.008, unpaired *t*-test) of V5-tagged Sox4 protein at the *TGFBR2* promoter relative to IgG control. **h** A 3.01-fold enrichment of V5 protein (*p* = 0.01, unpaired *t*-test) was observed at the *TGFBR2* enhancer in HCC1143^SOX4-V5^ cells relative to IgG control. Fold enrichment is normalized to PNOC promoter region, which is used as a negative control.

Given the dependency of SOX4 on TGFBR2 as a critical regulator of PI3K signaling, we next investigated the mechanism by which SOX4 regulates TGFBR2 expression. The SOX4 protein contains an HMG-box domain that enables it to interact with the A/TA/TCAAA/TG motif in the minor groove of the DNA to mediate transcription of its target genes^16,22^. Therefore, we first screened the genome upstream of the TGFBR2 transcriptional start site for potential functional genetic elements harboring the SOX4 binding motif. These analyses identified putative SOX4 binding motifs (AACAAAG) in the distal promoter (−2265 bp site) and enhancer regions (−8254 bp site) (Fig. 4a), suggesting that SOX4 may potentially bind and regulate *TGFBR2* expression through these conserved motif sequences.

Based on the identification of the putative binding sites, we next examined the capacity of SOX4 to bind to the *TGFBR2* promoter and enhancer by performing chromatin immunoprecipitation followed by quantitative-PCR (ChIP-qPCR) analysis. To circumvent the issue of antibody non-specificity and cross-reactivity during ChIP analysis, CRISPR/cas9 genome editing was used to add a V5 epitope tag to the carboxyl (C)-terminus of endogenous *SOX4* in HCC1143 cells (hereafter referred to as HCC1143^SOX4-V5^ cell line) (Fig. 4b and Supplementary Fig. 5a). DNA sequencing (Supplementary Fig. 5a), western blot analyses (Fig. 4c), and immunofluorescence microscopy (Fig. 4d) confirmed expression of the SOX4-V5 protein in HCC1143^SOX4-V5^ cells but not in the parental HCC1143 cell line. Finally, to ensure that the C-terminal epitope tag did not disrupt the observed effect of SOX4 on PI3K/Akt signaling and/or TGFBR2 expression, we examined the effect of siRNA-mediated silencing of SOX4 on these processes. SOX4 mRNA (Fig. 4e; *p* < 0.0001) and protein levels (Fig. 4f and Supplementary Fig. 5b, *p* = 0.002) were significantly depleted upon siRNA-mediated silencing of SOX4 and, as expected, V5 protein expression (Fig. 4f and Supplementary Fig. 5b, *p* = 0.001) was significantly reduced in HCC1143^SOX4-V5^ cells following siSOX4 treatment when compared to siControl-treated cells. Likewise, we observed a significant reduction in TGFBR2 protein expression (*p* = 0.031), which we have established as a downstream target of SOX4, as well as phosphorylated Akt (S473) (*p* = 0.01), P70S6K (Thr389) (*p* = 0.0007), and 4EBP1 (Thr37/46) (*p* = 0.01) proteins upon SOX4 inhibition (Fig. 4f and Supplementary Fig. 5b). These data indicate that addition of the V5 epitope tag to the C-terminal domain of endogenous *SOX4* does not interfere with these specific functions of the SOX4 protein in HCC1143^SOX4-V5^ cells, and suggest that the V5 epitope could be used as a marker to study SOX4 function in this cell line.

Based on these results, we next used the HCC1143^SOX4-V5^ cell line to examine the relative enrichment of SOX4-V5 on the *TGFBR2* promoter and enhancer. Chromatin Immunoprecipitation (ChIP) using an antibody against the V5 epitope was used to isolate V5-tagged SOX4. Quantitative RT-PCR primers spanning the SOX4 binding motif in the distal promoter and enhancer regions were then used to quantify levels of SOX4 at each site (Fig. 4a). ChIP-qPCR analysis revealed a greater than threefold significant enrichment (*p* = 0.008) of SOX4 at both the TGFBR2 promoter (Fig. 4g) and enhancer (*p* = 0.0098) regions relative to the IgG control (Fig. 4h).

### SMARCA4 and SOX4 form an essential regulatory complex at the TGFBR2 promoter and enhancer

**Fig. 5:**
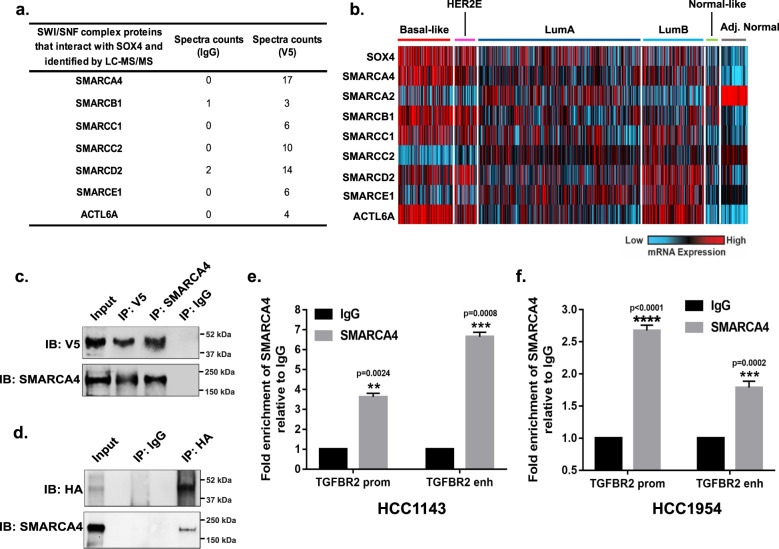
SOX4 and SMARCA4 form a complex at TGFBR2 promoter and enhancer region. **a** Potential interacting partners of SOX4 from the SWI/SNF chromatin remodeling complex family of proteins identified by LC-MS/MS. Spectral counts for IgG and V5 antibody for each protein is listed. **b** mRNA expression patterns of SWI/SNF complex core subunit genes relative to SOX4 were determined for 1031 human breast tumors and 94 adjacent normal samples from the TCGA dataset. Samples are organized by PAM50 molecular subtype; red indicates high mRNA expression, and blue depicts low mRNA levels. **c** Co- immunoprecipitation of SOX4 (V5) and SMARCA4 followed by reciprocal western blot analyses demonstrates the interaction between these proteins in HCC1143^SOX4-V5^ cells; results are representative of three independent experiments. **d** HEK293T cells were lentivirally overexpressed with HA-SOX4 and co-immunoprecipitation performed with HA antibody followed by western blot detection of SMARCA4 demonstrating the interaction between these proteins. **e** SMARCA4 ChIP-qPCR in HCC1143 demonstrated a 3.6- and 6.6-fold enrichment of SMARCA4 at the *TGFBR2* promoter (*p* = 0.0024; unpaired *t*-test) and enhancer (*p* = 0.0008; unpaired *t*-test) respectively. **f** SMARCA4 ChIP-qPCR in HCC1954 cells demonstrated a 2.7- and 1.8-fold enrichment of SMARCA4 at the *TGFBR2* promoter (*p* < 0.0001; unpaired *t*-test) and enhancer (*p* = 0.0002; unpaired *t*-test), respectively.

Regulation of gene expression is dependent on the ability of the cell to modulate the accessibility of the tightly condensed chromatin to sequence-specific transcription factors and basal transcription machinery^46,47^. Since SOX4 lacks the ability to remodel chromatin, we sought to identify its interaction with the cell-specific chromatin remodeling machinery and demonstrate the role of these co-factors in regulating expression of SOX4 target genes, specifically *TGFBR2*. To identify the SOX4 interactome, we immunoprecipitated SOX4 protein complexes from HCC1143^SOX4-V5^ cells using antibodies against the V5 epitope (or IgG control) followed by LC-MS/MS analysis. Our analyses identified 1700 proteins enriched in the V5 and IgG antibody pull-downs with a peptide FDR of <1% (Supplementary Data 5). Interestingly, these analyses identified several components of the SWI/SNF chromatin remodeling complex, including SMARCA4, SMARCB1, SMARCC1, SMARCC2, SMARCD2, SMARCE1, and ACTL6A as potential SOX4 co-factors (Fig. 5a). Given the recognized function of SWI/SNF complex as epigenetic regulatory proteins, and the noted contribution of several of these proteins to breast-cancer pathogenesis^48–50^, we postulate that SOX4 remodels the *TGFBR2* regulatory regions by interacting with the functional SWI/SNF chromatin remodeling complex.

To test this possibility, we first examined the subtype-specific expression of the identified SWI/SNF complex proteins in human breast tumors. Consistent with our previous studies, analyses of gene expression data from the TCGA cohort (*n* = 1031 tumors, *n* = 94 adjacent normal tissue) identified significant upregulation of *SOX4* expression in basal-like tumors (Fig. 5b)^5^. More importantly, our analyses identified increased expression of the core components of the SWI/SNF complex *SMARCB1*, *SMARCC1*, *SMARCD2,* and *ACTL6A* in basal-like tumors compared to other subtypes and adjacent normal tissue (Fig. 5b). These data suggest that the functional SWI/SNF complex may be aberrantly activated in basal-like tumors and cooperate with SOX4 to mediate its activity. Interestingly, however, *SMARCA4* and *SMARCA2*, which are the ATPase subunits of this complex, demonstrated an anti-correlated pattern of expression with basal-like tumors expressing high levels of *SMARCA4* mRNA but low levels of *SMARCA2* transcripts (Fig. 5b). Consistent with these observations, a significant positive correlation was identified between *SOX4* and *SMARCA4* (*r* = 0.4, *p* = 8.9 × 10^−36^), *SMARCB1* (*r* = 0.3, *p* = 1.4 × 10^−28^), *SMARCC1* (*r* = 0.4, *p* = 5.3 × 10^−42^), *SMARCD2* (*r* = 0.1, *p* = 1.5 × 10^−5^), and *ACTL6A* (*r* = 0.5, *p* = 2.3 × 10^−61^) gene expression in basal-like tumors (Fig. 5b). Conversely, a weak negative correlation was observed between SOX4 and SMARCA2 (*r* = −0.2, *p* = 3.5 × 10^−8^) mRNA expression in this subset of TCGA tumors (Fig. 5b). Similar gene expression and correlation patterns were also observed between *SOX4* and SWI/SNF core subunits in the METABRIC dataset (Supplementary Fig. 6a). Given that SMARCA4, SMARCB1, SMARCC1, SMARCD2, and ACTL6A are highly expressed in basal-like tumors in TCGA and METABRIC datasets, our data suggest that the functional SWI/SNF complex may be significantly upregulated in these tumors and may interact with SOX4 to contribute to the aberrant signaling activity apparent in these tumors.

In order to confirm the interaction between SOX4 and SMARCA4, which is the catalytic subunit of the SWI/SNF chromatin remodeling complex, we performed co-immunoprecipitation (Co-IP) experiments in HCC1143^SOX4-V5^ cells. As illustrated in Fig. 5c, SMARCA4 was detected by western blot analyses following immunoprecipitation of SOX4 using an antibody against the V5 epitope tag. Importantly, reciprocal Co-IP experiments using the anti-SMARCA4 antibody followed by western blot analysis confirmed this interaction (Fig. 5c). Finally, to validate these findings in an independent cell line, using an independent epitope tag, HA-tagged SOX4 was lentivirally overexpressed in 293T cells. SOX4 was immunoprecipitated using an HA-specific antibody followed by SMARCA4 western blot analysis, which confirmed the interaction between SOX4 and SMARCA4 (Fig. 5d).

To determine if SMARCA4 is present at the *TGFBR2* upstream regulatory regions, we analyzed publicly available SMARCA4 ChIP-seq data^51^ from MDA-MB-231 cells. Concurrently, we analyzed publicly available ChIP-seq data for histone modifications that mark the promoter (H3K4me3) and enhancer (H3K27ac) regions to further characterize the *TGFBR2* upstream regulatory regions^52^. These analyses identified increased enrichment of SMARCA4 at the *TGFBR2* promoter and enhancer regions, indicated by the increased enrichment of H3K4me3 and H3K27ac histone marks, respectively (Supplementary Fig. 6b). Interestingly, SMARCA4 enrichment coincided with the SOX4-bound loci at the *TGFBR2* promoter and enhancer regions that were previously identified (Fig. 4a) suggesting that SOX4 and SMARCA4 may form a complex at these sites. To validate this observation, SMARCA4 ChIP-qPCR analyses were performed in HCC1143 and HCC1954 cell lines using the previously described quantitative-PCR (qPCR) primers sets (Fig. 4). Our analyses demonstrated a significant 3.6-fold enrichment at the *TGFBR2* promoter (*p* = 0.0024) and 6.6-fold enrichment at the *TGFBR2* enhancer region (*p* = 0.0008) relative to IgG in HCC1143 cells (Fig. 5e). These results were confirmed in the HCC1954 cell line, which showed a 2.6-fold and 1.7-fold enrichment of SMARCA4 at the *TGFBR2* promoter (*p* < 0.0001) and enhancer (*p* = 0.0002) relative to IgG, respectively (Fig. 5f).

### SOX4 recruits SMARCA4 to regulatory regions to promote TGFBR2 expression

SMARCA4 containing SWI/SNF complexes demonstrate little sequence specificity in vitro and are required to interact with transcriptional activator proteins in order to be recruited to the regulatory regions of target genes^53,54^. Given that our data indicate that SMARCA4 forms a complex with SOX4 at *TGFBR2* regulatory regions, we next investigated whether SOX4 is required to recruit SMARCA4 to these regions.

**Fig. 6:**
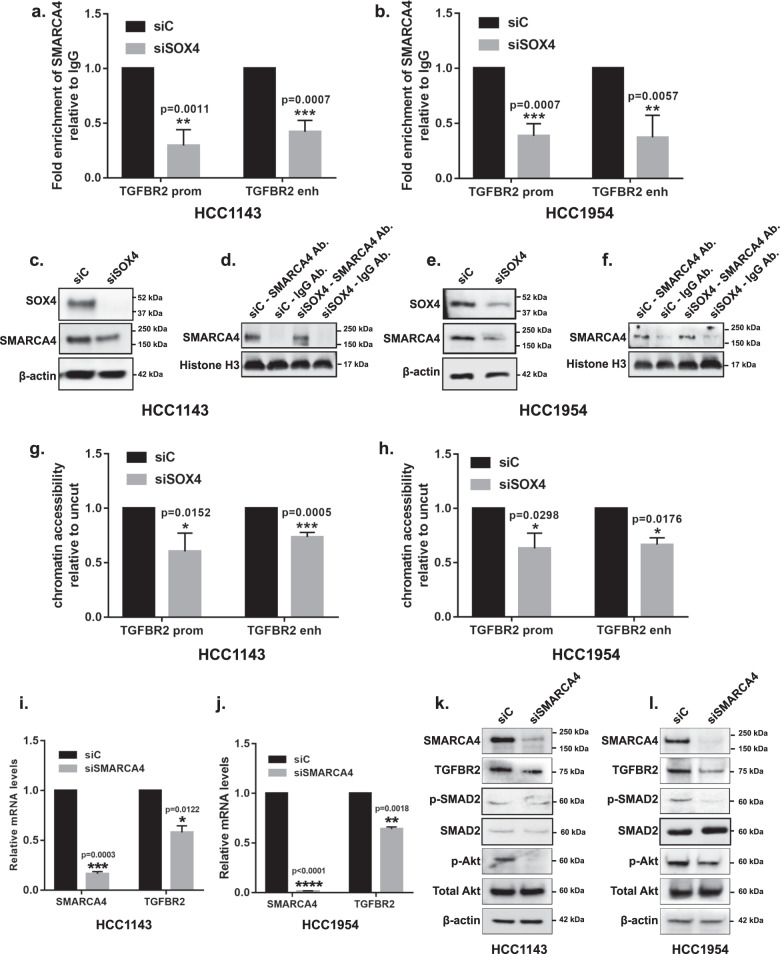
SOX4 recruits SMARCA4 to remodel chromatin and mediates TGFBR2 expression. ChIP-qPCR demonstrating reduction in SMARCA4 enrichment following siRNA-mediated knockdown of SOX4 by 70.49% (*p* = 0.001; unpaired *t*-test) and 57.9% (*p* = 0.0007; unpaired *t*-test) in HCC1143 (**a**) and by 61.49% (*p* = 0.0007; unpaired *t*-test) and 62.8% (*p* = 0.006; unpaired *t*-test) in HCC1954 (**b**) cells at the *TGFBR2* promoter and enhancer regions relative to the scrambled control. Western blot analysis demonstrating siRNA-mediated silencing of SOX4 results in decreased SMARCA4 protein expression in HCC1143 (**c**) and HCC1954 (**e**) cell lines. ChIP–western blot analysis demonstrating equivalent levels of SMARCA4 protein pulldown in cells treated with either siRNA targeting SOX4 or control siRNA in HCC1143 (**d**) and HCC1954 (**f**) cell lines. Chromatin accessibility assay demonstrating that accessibility was reduced following siRNA-mediated knockdown of SOX4 by 39.7% (*p* = 0.015; unpaired *t*-test) and 26.5% (*p* = 0.0005; unpaired *t*-test) in HCC1143 (**g**) and by 36.9% (*p* = 0.03; unpaired *t*-test) and 33.4% (*p* = 0.017; unpaired *t*-test) in HCC1954 (**h**) cells at the *TGFBR2* promoter and enhancer regions, respectively, relative to the scrambled control. qRT-PCR analysis demonstrating decreased TGFBR2 mRNA expression in HCC1143 (**i**) and HCC1954 (**j**) cell lines following RNAi-mediated silencing of SMARCA4. Representative western blot analysis demonstrating loss of TGFBR2 and pAkt protein expression in HCC1143 (**k**) and HCC1954 (**l**) cell lines following RNAi-mediated silencing of SMARCA4.

To investigate this hypothesis, SMARCA4 ChIP-qPCR analyses were performed in the presence and absence of SOX4 in HCC1143 and HCC1954 cells. Our analyses determined that siRNA-mediated depletion of SOX4 in HCC1143 cell line resulted in a significant reduction in SMARCA4 enrichment on both the *TGFBR2* promoter (70.39% reduction, *p* = 0.001) and enhancer (57.92% reduction, *p* = 0.0007) regions relative to the scrambled control (Fig. 6a). As expected, similar results were observed in HCC1954 cells which showed a 61.5% decrease of SMARCA4 at the *TGFBR2* promoter (*p* = 0.0007) and 62.8% decrease at the *TGFBR2* enhancer (*p* = 0.006) (Fig. 6b). We validated these findings in HCC1143 and HCC1954 cell lines which have been engineered to stably express one of two independent tet-inducible shRNA against SOX4. As illustrated in Supplementary Fig. 7, treatment with dox significantly abrogated the enrichment of SMARCA4 on the *TGFBR2* promoter in both sh-1 (73.6% reduction, *p* = 0.0002) and sh-2 (56.1% reduction, *p* = 0.0005) expressing HCC1143 cells (Supplementary Fig. 7a) as well as sh-1 (66.5% reduction, *p* = 0.0014) or sh-2 (56.0% reduction, *p* = 0.001) expressing HCC1954 cells (Supplementary Fig. 7b). Similar results were observed for SMARCA4 enrichment at the *TGFBR2* enhancer region with sh-1 expressing HCC1143 cells showing a 66.2% reduction, (*p* = 0.0004) and sh-2 expressing cells showing a 62.3% reduction (*p* = 0.002) relative to untreated cell lines (Supplementary Fig. 7a). Likewise, sh-1 (52.3% reduction, *p* = 0.02) or sh-2 (60.6% reduction, *p* = 0.002) expressing HCC1954 cells showed a similar significant reduction in SMARCA4 enrichment at the *TGFRB2* enhancer relative to untreated samples (Supplementary Fig. 7b).

Since previous studies have demonstrated that SOX4 can regulate SMARCA4 expression in MRC-5 fibroblasts cells^55^, these data suggested the possibility that the observed decrease in SMARCA4 recruitment at the TGFBR2 promoter may be due to decreased SMARCA4 protein expression following SOX4 silencing. Given the noted tissue-specific nature of SOX4 gene expression programs, we next assessed whether SOX4 could mediate expression of SMARCA4 in TNBC cell lines^56^. As illustrated in Fig. 6c (HCC1143) and Fig. 6e (HCC1954), siRNA-mediated silencing of SOX4 resulted in decreased SMARCA4 protein expression in both cell line models. To determine if the observed decrease in SMARCA4 expression was sufficient to explain the loss of SMARCA4 binding to the TGFBR2 regulatory regions, we performed ChIP followed by western blot analysis in siControl- and siSOX4-treated cells. These analyses detected equivalent SMARCA4 levels in both cell lines (Fig. 6d, f) demonstrating that an equal amount of chromatin bound SMARCA4 could be detected following siSOX4 or siControl treatment. These data indicate that while loss of SOX4 decreases overall SMARCA4 expression, it does not globally affect its ability to bind to DNA. Importantly, however, loss of SOX4 does affect SMARCA4 recruitment to the TGFBR2 promoter/enhancer indicating that this process is dependent on the presence of SOX4 at these regions. Moreover, these analyses also suggest that SOX4 mediates SMARCA4 activity at both the transcript and protein levels and may further explain why there is a strong correlation between SOX4 and SMARCA4 expression in human tumors (Fig. 5b and Supplementary Fig. 6a).

Next, we examined whether SOX4-mediated recruitment of SMARCA4 to the *TGFBR2* promoter and enhancer region is a silent or a functional recruitment. SMARCA4 is the catalytic subunit of the SWI/SNF complex and is required for chromatin remodeling to increase accessibility at genomic loci^47^. To assess the remodeling at the *TGFBR2* regulatory regions mediated by the SOX4-SMARCA4 interaction, we performed a micrococcal nuclease (MNase) digestion assay followed by qPCR^48^ following siRNA or shRNA depletion of SOX4. We determined that siRNA-mediated silencing of SOX4 resulted in a significant 39.7% (*p* = 0.015) and 26.5% (*p* = 0.0005) reduction in chromatin accessibility the *TGFBR2* promoter and enhancer regions, respectively, relative to the scrambled control in HCC1143 (Fig. 6g). A similar 36.9% (*p* = 0.03) and 33.4% (*p* = 0.018) reduction in chromatin accessibility was observed at the promoter and enhancer regions in HCC1954 cells following siRNA-mediated depletion of SOX4 (Fig. 6h). As expected, comparable results were observed after dox induced knockdown of SOX4 at the *TGFBR2* promoter in sh-1 (37.1% reduction, *p* = 0.03) or sh-2 (35.6% reduction, *p* = 0.004) expressing HCC1143 (Supplementary Fig. 7c) as well as sh-1 (82.1% reduction, *p* = 0.03) or sh-2 (99.8% reduction, *p* < 0.0001) expressing HCC1954 cells (Supplementary Fig. 7d). Similarly, we observed a 27.2% and 29.3% reduction in chromatin accessibility at the *TGFBR2* enhancer in sh-1 (*p* = 0.022) or sh-2 (*p* = 0.02) expressing HCC1143 cells, respectively, relative to untreated cells (Supplementary Fig. 7c). Likewise, sh-1 (59.6% reduction, *p* = 0.01) or sh-2 (95.1% reduction, *p* < 0.0001) expressing HCC1954 cells showed a significant reduction in chromatin accessibility at the *TGFRB2* enhancer relative to control cells (Supplementary Fig. 7d).

Collectively, these data suggest that SOX4 is required to recruit SMARCA4 to *TGFBR2* regulatory regions and that SMARCA4 is essential for chromatin remodeling and activation of *TGFBR2* expression. Consistent with the latter premise, qRT-PCR and western blot analysis demonstrated a significant reduction of TGFBR2 mRNA (HCC1143, *p* = 0.012; HCC1954, *p* = 0.001) and protein (HCC1143, *p* = 0.0009; HCC1954, *p* < 0.0001) levels following siRNA-mediated silencing of *SMARCA4* in HCC1143 (Fig. 6i, k and Supplementary Fig. 7f) and HCC1954 (Fig. 6j, l and Supplementary Fig. 7g) cell lines. As expected, Akt (Ser473) phosphorylation was significantly reduced in HCC1143 (Fig. 6k and Supplementary Fig. 7f) (*p* = 0.0037) and HCC1954 (Fig. 6l and Supplementary Fig. 7g) (*p* = 0.0002) cells following siSMARCA4 treatment compared to siControl-treated cells. These data suggest that SOX4-mediated recruitment of SMARCA4 on *TGFBR2* regulatory regions results in a more open and permissive chromatin conformation, resulting in increased TGFBR2 expression and downstream activation of PI3K signaling.

## Discussion

TNBC accounts for 10–15% of new breast-cancer cases and ~1-in-4 breast-cancer-related deaths each year^1,2^. Although a number of recent advances have improved treatment strategies, there is a clear need to better understand the mechanisms driving TNBC genesis and progression in order to develop more effective therapies. Despite the fact that basal-like or triple-negative breast tumors are characterized by aberrant activation of PI3K/Akt signaling, PI3K-family inhibitors have shown limited clinical success either as single agent therapies or as combination therapies in this subset of patients^7,12,14^. The lack of clinical response can be attributed to a number of factors, including, but not limited to, the development of adaptive resistance mechanisms and/or activation of parallel signaling pathways^7,12,14^. These previous studies suggest that understanding and targeting additional mechanisms of the PI3K pathway activation and/or complementary pathways will be important for optimizing therapeutic strategies for TNBC patients. While several large-scale genomic studies have identified copy-number alterations or mutations in key regulatory components of the PI3K pathway in specific subsets of TNBC or basal-like tumors^3^, there is a significant need to understand additional mechanisms regulating PI3K signaling and to identify key genes that mediate the development of adaptive responses to PI3K pathway inhibitors in these patients.

**Fig. 7:**
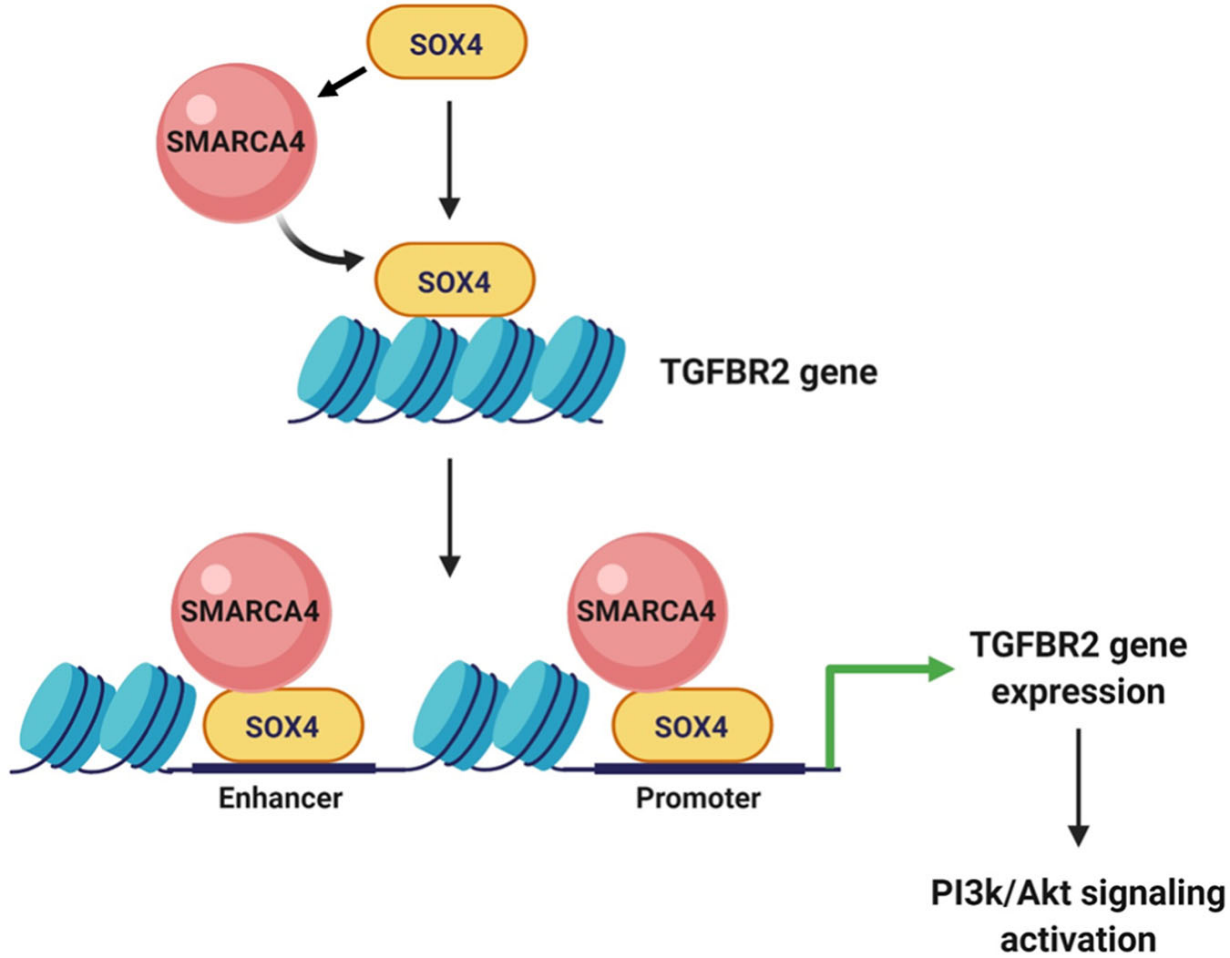
The Sox4-SMARCA4 complex mediates PI3K/Akt signaling by regulating TGFBR2 gene expession. Schematic representation of the hypothetical model demonstrating SOX4 and SMARCA4-mediated regulation of the TGFBR2 gene expression and activation of PI3K/Akt signaling.

A recent genomics-based study of human tumors from our laboratory identified *SOX4* as a potential driver of PI3K/Akt signaling in basal-like breast cancer and in vitro studies confirmed that SOX4 can regulate this pathway in basal-like cell lines^5^. In the current study, we identified a novel mechanism by which SOX4 regulates PI3K signaling in basal-like breast cancer. Using a combination of proteomics-based kinome profiling, transcriptomic analyses and molecular approaches, we identified TGFBR2 as a critical downstream target of SOX4 in basal-like breast-cancer cell lines. Importantly, in vitro analyses confirmed that SOX4-mediated activation of PI3K signaling is regulated by TGFBR2 activity. Mechanistic studies established direct binding of SOX4 to the *TGFBR2* promoter and enhancer regions indicating that TGFBR2 is a direct transcriptional target of SOX4. Finally, we demonstrated that SOX4 recruits and forms a complex with the SMARCA4 at the *TGFBR2* promoter and enhancer regions. This complex is required to mediate chromatin remodeling and facilitate activation of *TGFBR2* expression and downstream PI3K signaling (Fig. 7). Therefore, the current study not only delineates a novel mechanism by which SOX4 cooperates with SMARCA4 to regulate PI3K signaling through TGFBR2 expression but these findings also have a potentially broader implication for regulation of oncogenic signaling and resistance mechanisms in TNBC.

SOX4 is a well-established oncogene and a member of SOX C family of SRY-related HMG-box (SOX) transcription factors^16^. Increased *SOX4* expression has been shown to be associated with malignant transformation and metastasis in several cancer types^21–24^, including breast cancer^5,17–20^. Despite these findings, a limited understanding of the mechanisms, including gene expression programs mediated by SOX4 and necessary co-factors, have been defined in basal-like or TNBCs. Analyses of human breast tumors indicate that *SOX4* mRNA is uniformly overexpressed in basal-like tumors relative to adjacent normal tissue along with the SWI/SNF catalytic subunit *SMARCA4* and *SMARCB1 SMARCC1, SMARCD2*, and *ACTL6A*, which encode for the essential subunits of this complex (Fig. 5b). Our data further demonstrate that SMARCA4 and SOX4 form a novel complex to mediate TGFBR2 expression and PI3K signaling and that inhibition of either gene results in a similar effect on downstream signaling. These results are consistent with the reported pro-oncogenic role for SWI/SNF complex proteins, and in particular the role of SMARCA4 in regulating oncogenic phenotype in breast cancer^28,29,34,35,48,57^. As such, our data indicate that the SOX4-SMARCA4 complex may play an integral role in TNBC genesis and progression by activation of novel gene expression programs in this subset of breast tumors.

TGFBR2 is a transmembrane serine/threonine protein kinase that activates canonical SMAD signaling following hetero-dimerization with TGFBR1 in a ligand-dependent or independent manner^58^. While, it is unclear whether SOX4 activation of PI3K signaling is ligand-dependent, it has been documented that the TNBC tumor microenvironment is often enriched with TGFβ-family ligands produced by tumor cells and/or tumor-associated stromal and immune cells^59,60^. However, since aberrantly, or constitutively, activated TGFBR2 can also modulate TGFβ signaling independent of ligand, additional studies will be required to further delineate this process in SOX4 overexpressing human tumors. Nonetheless, the impact of TGFβ signaling on breast-cancer genesis and progression, including its role in EMT and proliferation, has been well-documented^17,19,20^. In addition to canonical signaling, TGFBR2 can also regulate noncanonical signaling through activation of PI3K, MAPK, and WNT signaling^61^. While previous studies have indicated that cross-talk between SOX4 and TGFβ can mediate activation of canonical Smad signaling in order to induce EMT^17,19,20^, the role of SOX4 in regulating noncanonical TGFβ signaling in cancer, or in TNBC or basal-like breast cancer specifically, has not been reported. TGFBR2 has been shown to stimulate noncanonical PI3K signaling by interacting with and phosphorylating the p85 regulatory subunit of PI3 kinase in fibroblast cell lines^62^. Therefore, the observation that SOX4 and SMARCA4 mediate PI3K activity through TGFBR2 expression identifies a novel and direct role for TGFBR2 in activating noncanonical PI3K signaling in basal-like breast cancer. Furthermore, these findings may also suggest that additional oncogenic signaling pathways, downstream of TGFβ, may be activated by the SOX4/SMARCA4 complex and contribute to tumor development and progression. Consistent with this idea, previous studies have indicated that increased TGFBR2 expression is associated with resistance to ALK and EGFR inhibitors in non-small cell lung carcinoma. In these studies, TGFBR2 was shown to mediate resistance by activating noncanonical MEK-ERK signaling^63^. Similarly, in breast cancer, autocrine activation of TGFBR2 expression in response to chemotherapy has been reported to regulate proliferation of breast-cancer stem cell populations and contribute to tumor recurrence^59^. Given that our data identifies SOX4 and SMARCA4 as key modulators of TGFBR2 expression and PI3K activity in basal-like breast cancer, it remains to be determined if aberrant TGFBR2 activity can also regulate a similar resistance mechanism to PI3K-family inhibitors in TNBC patients.

Profiling of the drug-able kinome by MIB/MS analysis identified a number of kinases that were significantly depleted or activated following SOX4 knockdown. Importantly, TGFBR1, which forms a functional complex with TGFBR2, and EPHA4, a known downstream target of TGF-β signaling that is associated with poor prognosis in basal-like breast-cancer patients^64^, were downregulated in MIB/MS analysis. These findings along with depleted p-SMAD2 protein levels upon SOX4 knockdown indicates that SOX4-mediated activation of TGFBR2 could potentially regulate both canonical and noncanonical TGF-β signaling pathways. Moreover, in addition to TGFBR2 and EPHA4, we identified several kinases (BMPR1A, ACVR1B, LCK, and EGFR), which have been shown to activate PI3K activity to be downregulated upon SOX4 knockdown, suggesting that SOX4 may mediate activation of PI3K/Akt signaling through multiple mechanisms and/or may mediate complementary pathways that contribute to resistance to PI3K-family drugs. Consistent with this premise, MIB/MS analyses also identified significant enrichment of MAPK signaling pathway components MAPK1, MAP2K1 (MEK1), and MAP2K4 (MEK4) following siRNA-mediated SOX4 silencing. Activation of MAPK signaling has been identified as a feedback mechanism to compensate for the downregulation of PI3K signaling in breast-cancer patients treated with everolimus in ER+ breast cancers^65^. This suggests that enrichment of these MAP kinases could function as compensatory mechanism to PI3K inhibition and dual targeting of these pathways may yield a more durable response in TNBC patients. In agreement with this notion, a phase 1 clinical trial investigating co-targeting of PI3K and MAPK signaling pathways showed a durable response in KRAS-driven tumors of colon, pancreas, skin, and lung^66^, suggesting that targeting these pathways together may prove beneficial in TNBC patients. Moreover, several clinical trials are currently underway to test the efficacy of targeting these pathways together in TNBC patients^67^. Taken together, these data in combination with transcriptome profiling by RNAseq, the identification of the SOX4 interactome by quantitative MS/MS and SOX4-mediate kinome by MIB/MS analysis suggest that additional signaling pathways that contribute to aspects of tumor progression may be regulated by SOX4 alone or in concert with SMARCA4 in basal-like tumors.

The current study has identified a novel mechanism by which PI3K signaling is regulated in basal-like or TNBC. Our data demonstrate that SOX4 and SMARCA4, which are uniformly overexpressed in basal-like tumors, form a previously unreported complex that is required to mediate epigenetic reprogramming of the TGFBR2 chromatin landscape and regulate TGFBR2 expression to promote PI3K signaling in this subset of breast-cancer patients. Profiling of the drug-able kinome in addition with transcriptome analyses suggest that SOX4 alone and in combination with SMARCA4 may modulate unique gene expression programs that contribute to breast-cancer genesis, progression, and response to therapy. Additional studies will be required to delineate these signaling networks, their impact on tumorigenesis and response to therapies, including resistance to PI3K-family inhibitors in TNBC patients, and to determine whether these networks are active in other breast-cancer subtypes and/or other forms of cancer.

## Methods

### Gene expression analysis of human breast tumors and breast-cancer cell lines

RNA sequencing data from human tumors (*n* = 1031) and adjacent normal tissue (*n* = 94) were acquired from the TCGA data portal (https://tcga-data.nci.nih.gov/tcga/), RSEM values were log-transformed and median-centered, and genes were filtered to exclude those that were not present in at least 80% of samples^68^. Illumina HT-29 v3 expression data for the METABRIC project (*n* = 1992) was acquired from the European Genome-phenome Archive at the European Bioinformatics Institute (https://www.ebi.ac.uk/ega/) and data were median-centered^36^. PAM50 classification has been previously reported for the TCGA and METABRIC datasets; these data are summarized in Supplementary Data 1 and 2^5,36^. The PI3K pathway gene expression signature score was calculated for each tumor sample in the TCGA and METABRIC dataset as previously reported^39^. Briefly, gene expression data for each dataset were filtered to those genes present in the PI3K signature and the pathways score determined for each sample by calculating the mean value of the signature genes. The signature score is reported for each sample in each dataset (Supplementary Data 1 and 2). To examine the relationship between *SOX4* mRNA expression and PI3K activity, samples in each dataset were delineated into quartiles, and PI3K pathway score in the top and bottom quartiles was assessed by a two-tailed *t*-test. Relative *SOX4* expression between basal-like tumors (*n* = 179) and adjacent normal (*n* = 94) samples from the TCGA dataset was compared by a two-tailed *t*-test. For analyses of individual gene expression patterns relative to PAM50 subtype, expression values for specific genes were selected from the TCGA or METABRIC dataset. Samples were categorized by PAM50 subtype, differences in subtype-specific expression determined by ANOVA test followed by Tukey test for pairwise comparison, and relative expression patterns visualized by the “imagesc” function in MATLAB. To identify basal-like breast-cancer cell lines with high SOX4 expression for mechanistic studies, SOX4 mRNA and protein expression was assessed in a panel of basal-like cell lines. Affymetrix U133+2 gene expression data for a panel of 24 basal-like breast-cancer cell lines were acquired from Gene Expression Omnibus (GEO) (GSE12777)^40^, data were MAS5-normalized and log2-transformed using Affymetrix Gene Expression Console, and gene-specific expression probes were collapsed to the median using GenePattern^69^ and *z*-score reported. MS-derived protein expression values for nine basal-like breast-cancer cell lines were acquired from the CCLE project^70^ and *z*-score reported.

### Cell culture and shRNA/siRNA knockdown

Breast-cancer cell lines and HEK293T cells were purchased from the American Tissue Culture Collection (Manassas, VA, USA) and cultured according to the suggested guidelines. HCC1143 or HCC1954 cell lines expressing one of the two tetracycline (tet)-inducible shRNA expressing cell lines were created using the pTRIPZ Inducible Lentiviral shRNA system (GE Dharmacon). The catalog number for shRNA (1) is: V2THS_153343 and for shRNA (2) is: V3THS_401094. The shRNA expression was induced using 2.0 µg/mL of dox for HCC1143 and 1.0 µg/mL of dox for HCC1954. Silencing of SOX4 expression was verified by qRT-PCR analysis. For siRNA knockdown experiments, cells were subcultured at 60–70% confluence before transfections. Lipofectamine RNAiMAX (ThermoFisher) was used to transfect cells with 50 nm of SMART pool siRNA targeting either SOX4 (L-011779-00-0005), SMARCA4 (L-010431-00-0005), TGFBR2 (LQ-003930-00-0005), or non-targeting siRNA control pool (D0012061305) according to manufacturer’s instructions (Dharmacon) for 96 h prior to harvesting cells for subsequent experiments.

### RNA extraction and quantitative real-time PCR

Total RNA was isolated using the RNeasy plus Mini Kit (Qiagen), and cDNA was synthesized using the QuantiTect Reverse Transcription kit (Qiagen). qPCR was performed and analyzed using Applied Biosystems QuantStudio3 real-time thermal cycler system. Primer sequences are as follows: human SOX4: Forward: 5′-CTCTCCAGCCTGGGAACTATAA-3′, Reverse: 5′-CGGAGGTGGGTAAAGAGAGAA-3′; human SMARCA4: Forward: 5′- AGTGCTGCTGTTCTGCCAAAT −3′, Reverse: 5′-GGCTCGTTGAAGGTTTTCAG-3′; human TGFBR2: Forward: 5′- AAATGGAGGCCCAGAAAGAT-3′, Reverse: 5′- ACTTGACTGCACCGTTGTTG-3′; and human GAPDH: Forward: 5′- TCTGACTTCAACAGCGACAC-3′, Reverse: 5′-CCAGCCACATACCAGGAAAT-3′.

### Cell extracts and western blot analysis

Cells were harvested using Triton lysis buffer (TLB) containing 25-mM HEPES, 100-mM NaCl, 1-mM EDTA, 10% glycerol, and 1% Triton X-100 with protease and phosphatase inhibitor added fresh prior to use. Protein concentrations were measured using the BCA protein assay kit and 35–50 µg of protein was loaded on 4–20% Mini-protean TGX gradient gel (BioRad) at 100 V for 2 h at room temperature and transferred onto nitrocellulose membrane at 35 V for 2 h at 4 °C. The membranes were blocked using AdvanBlock-chemi blocking solution (Advansta) for 1 h at room temperature, incubated with primary antibody (1:1000) overnight at 4 °C followed by incubation with HRP-conjugated secondary antibodies (1:2000, Cell Signaling Technology) for 1 h at room temperature. The signal was developed using SuperSignal West Pico Chemiluminescent Substrate (ThermoFisher Scientific) and digitally imaged using the ChemiDoc Touch Imaging System (BioRad). Primary antibodies against pAkt (S473) (4060S), Total Akt (9272S), pP70S6K (Thr389) (9205S), P70S6K (9202S), p4EBP1 (Thr37/46) (2855S), 4EBP1 (9452S), p-SMAD2 (3108S), SMAD2 (3122 S), V5 (13202S), HA (3724S), β-actin (4970S) were purchased from Cell Signaling Technology. The antibodies against SOX4 (PB9618, BosterBio), SMARCA4 (sc-374197, Santa Cruz Biotechnology), and TGFBR2 (AF-241-SP, R&D Biosystems) were used according to the instructions of the manufacturer. All western blots presented in a given figure panel are derived from a single experiment and the full uncropped images are presented in Supplementary Fig. 8; a minimum of three independent experimental replicates were performed for each analysis.

### CRISPR-Cas9-mediated tagging of SOX4 locus with V5 epitope

The SOX4 locus was tagged with a V5 epitope at the C-terminus in HCC1143 cells using CRISPR-Cas9-mediated knock-in strategy. Briefly, a duplex oligo containing gRNA C187 was cloned into pSpCas9 (BB)-2A-GFP (pX458), which was a gift from Dr Feng Zhang (Addgene # 48138) to create plasmid pX458-C187. The donor oligo containing the V5 tag sequence was ordered from IDT, PAGE purified, and 0.5 µg of donor oligo was co-transfected with 2 µg of pX458-C187 into 2 × 106 HCC1143 cells using the NEON transfection system. Twenty-four hours post-transfection, 0.5 × 106 GFP-positive cells were individually sorted into seven 96-well plates from which 32 clones grew and were genotyped using the following primers, SOX4A: 5′-AAGACGACCTGCTCGACCTGAAC-3′; SOX4B: 5′-CTTGATCCGACGACGAGAACGC-3′. Ten clones were sequenced by the Sanger method and two clones, which had a complete V5 tag inserted correctly at the C terminus, were expanded into cell lines. The gRNA and oligo donor sequence used are listed below:

gRNA sequence and location:

CGAGTCCAGCATCTCCAACC TGG [GRCh38.p12 Chrom.6: 21595923–21595945(+)]

Donor oligo sequence [(-) strand]:

5′…TCACTTTTTTTTTCTCCTCTCCTACCCCCCCCGGCCCTTCTCCCTGCCTGCGCGCCCTtcaCGTAGAATCGAGACCGAGGAGAGGGTTAGGGATAGGCTTACCGTAGGTGAAAACGA**GGTTGGAGATGCTGGACTCG**AGCCAGTCTCCCGAGATCATCTCGCTCAC…3′

V5 tag, underlined; altered PAM motif, double underlined; stop codon, lower case; gRNA sequence, bold.

### Immunofluorescence

Immunofluorescence studies were performed as described previously^5^. Briefly, cells were grown on coverslips, fixed with 4% paraformaldehyde in phosphate-buffered saline (PBS), and permeabilized with 0.25% Triton X-100 in PBS for 8 min at room temperature. Cells were blocked in 1% BSA in PBS with 0.05% Tween 20 for 45 min at room temperature, incubated with V5 antibody for 1 h at room temperature, and then incubated with fluorochrome-labeled secondary antibody for 1 h at room temperature. The coverslips were counterstained with DAPI and imaged with a Nikon Eclipse TE-2000U fluorescent microscope.

### Co-IP and LC-MS/MS analysis

The HA epitope-tagged SOX4 plasmid was generously provided by Dr Carlos Moreno (Emory University) and purified to generate lentiviral particles for transduction of HEK293T cells. Protein lysates were prepared from either HCC1143^SOX4-V5^ or HA-SOX4-transduced HEK293 cells and Co-IP studies were performed as described previously^71^. Briefly, cells were washed two times with PBS and lysed in TLB buffer using diagenode bioruptor. Lysate was then centrifuged at 13,000 × *g* for 15 min. Protein concentrations were measured using the BCA protein assay kit and 2000 µg of protein lysate was incubated with 5 µg of antibody against HA, V5, or SMARCA4, or with a non-specific IgG antibody overnight at 4 °C, followed by the addition of protein G Dynabeads (Thermofisher #10004D) and an additional incubation while rocking for 2 h at 4 °C. Beads were washed three times in the lysis buffer, eluted with 2X SDS gel loading buffer, and analyzed by western blotting.

For LC-MS/MS analysis, 9000 µg of HCC1143 SOX4-V5 protein lysate was used to precipitate the protein complexes using either 5 µg of V5 antibody or a non-specific IgG control as described above. The precipitated protein complexes were separated on a 4–20% gradient SDS gel, visualized using the SYPRO Ruby protein gel staining kit (Thermofisher #S12000) and analyzed on Thermo Scientific Orbitrap Fusion Lumos Tribrid mass spectrometer. Briefly, each gel lane was cut, in-gel trypsin digested (protein/trypsin ∼50:1) at 37 °C for 16 h. The tryptic peptides were extracted and desalted using a C-18 cartridge prior to LC-MS/MS analysis. For LC-MS/MS, the peptides were separated on a C-18 nano column (75 μm × 50 cm, 2 μm, 100 Å) at a flow rate of 300 nL/min on an UltiMate 3000 LC system using a 2 h gradient of solvent A (2% acetonitrile in 0.1% FA) and solvent B (85% acetonitrile in 0.1% FA). The MS/MS spectra were acquired on an Orbitrap Fusion Lumos mass spectrometer (Thermo Scientific, Canoga Park, CA) using the data-dependent analysis mode. MS scan range is *m/z* 375–1500 with the resolution of 120,000 (FWHM). The capillary temperature was set to 275 °C, and the spray voltage set to 2 kV. The MS/MS spectra were searched against the Uniprot human database (74,072 entries) using the Sequest search engine through the Proteome Discoverer (Version 2.3) platform. The mass tolerance was 10 ppm for MS and 0.1 Da for MS/MS. The variable modifications included methionine oxidation and protein N-terminus acetylation, whereas the fixed modification included cysteine carbamidomethylation. All proteins and peptides were identified with a false discovery rate of <1%. Relative quantitation was calculated based on a spectral counting method, where the spectral counts of identified proteins in V5 pulldown were divided by their corresponding spectral counts in non-specific IgG control. To avoid a large ratio from the presence of zero in the denominator, we arbitrarily added two spectral count to both the numerator and denominator during the ratio calculation.

### TGFBR2 overexpression and rescue experiments

pHAGE-TGFBR2 plasmid was a gift from Drs Gordon Mills and Kenneth Scott (Addgene plasmid # 116800). Lentiviral particles were generated by co-transfecting pHAGE-TGFBR2 plasmid and the packaging plasmids (psPAX2 and pMD2.G) in 293T packaging cell line using Lipofectamine 2000 transfection reagent. Viral supernatants were collected at 48 and 72 h, centrifuged to remove cell debris, filtered through 0.45-μm filters (Corning) and viral particles quantified using the Lenti-X GoStix Plus kit. The purified pHAGE-TGFBR2 viral particles were then used to perform overexpression and rescue experiments by infecting (MOI = 3) HCC1143 and HCC1954 cells that have been transfected with siRNA targeting either SOX4 or non-targeting control siRNA for 96 h. Cells were harvested for protein analysis 48 h post overexpression.

### Kinome profiling by MIB/MS

For MIB affinity chromatography, HCC1143 cells treated with either siRNA targeting SOX4 or non-targeting control siRNA for 96 h were washed 1X with cold PBS, lysed in 50-mM HEPES, 150-mM NaCl, 0.5% Triton X-100, 1-mM EDTA, 1-mM EGTA, at pH 7.5 containing 10-mM NaF, 2.5-mM NaVO4, Complete protease Inhibitor Cocktail (Roche), and 1% Phosphatase Inhibitor Cocktails 2 and 3 (Sigma). Lysate was sonicated three times (10 s) on ice and centrifuged (10 min, 13,000 rpm) at 4 °C and the supernatant syringe-filtered through a 0.2-mM SFCA membrane. Lysate was equalized at 500-µg total protein per experiment to 1-M NaCl and flowed over kinase inhibitor bead resin, a 175-μL mixture of five kinase inhibitors (VI-16832, PP58, Purvalanol B, UNC-21474, and BKM-120) custom-synthesized with hydrocarbon linkers (except Purvalanol B) and covalently linked to ECH-Sepharose (or EAH-Sepharose for Purvalanol B) beads as previously described^41^, followed by 30 volumes of washes with high salt (1-M NaCl) and low salt (150-mM NaCl) buffer (50-mM HEPES, 0.5% Triton X-100, 1-mM EDTA, 1-mM EGTA, at pH 7.5). Bound kinases were eluted by boiling with 0.5% SDS and 1% β-mercaptoethanol in 100-mM Tris-HCl, pH 6.8, 2 × 15 min, treated with DTT (5 mM, 25 min at 60 °C) and Iodoacetamide (20 mM, 30 min in the dark at RT), and spin-concentrated to 100 μL (Amicon Millipore Amicon Ultra-4, 10-K cutoff) before Methanol/Chloroform precipitation. Proteins were trypsinized overnight at 37 °C, extracted with water-saturated ethyl acetate four times, then dried down in a speed-vac. Peptides were desalted with C-18 spin columns (Cat. 89870, Pierce) according to the manufacturer’s instructions.

Peptides were resuspended in 0.1% formic acid. Forty percent of the final peptide suspension was injected onto a 5-cm PepMap trap column and then a Thermo Easy-Spray 75 μm × 25 cm C-18 column and separated on a 110-min gradient (3–28% ACN), B was increased to 40% over 2 min, up to 90% over 2 min, held at 90% for 2 min, and decreased back to 3% using an Ultimate 3000. The Thermo Q Exactive Plus MS ESI parameters were as follows: 350–1700 *m/z*, 3e6 AGC MS1, 100-ms MS1 max inject time, 1e5 AGC MS2, 17,500 resolutions, 75-ms MS2 max inject time, 15 loop count, 1.8-*m/z* isolation window, NCE 27%, 30-s dynamic exclusions, excluding unassigned, 1, 8, and >8 charge states. Thermo MS raw files were processed for label-free quantification by MaxQuant LFQ (MaxQuant version 1.6.10.43) with default parameters, using a Uniprot/Swiss-Prot human database, fixed carbidomethyl (C) and variable oxidation (M), and Acetyl (Protein N-term) modifications. Matching between runs was enabled with a match time window of 3 min. Kinase LFQ intensities with two or more unique + razor peptides were processed using Perseus (version 1.6.10.50)—LFQ intensities were log_2_-transformed and if three values were present in at least one group (siControl or siSOX4), missing values were imputed by column using default parameters. Unpaired Student’s *t* test was performed in Perseus to report log_2_ fold change and the associated *p* value.

### Next-generation RNA sequencing and data analysis

Total RNA was extracted from HCC1143 cells treated with either siRNA targeting SOX4 or non-targeting control siRNA for 96 h. RNA libraries were prepared using the NuGen Ovation Universal RNA-Seq System (Catalog # 0343–32) and paired-end (2 × 48 bp) sequencing was performed using the Illumina NextSeq system. Paired-end FASTQ files were processed using default parameters of Kallisto version v0.43.1, with 100 bootstraps for the expectation-maximization algorithm. Kallisto transcriptome index was built using the Ensembl human genome reference build 38. Gene level summarization of Kallisto abundance (TPM) was established using the R package Tximport v1.0.3 and R v3.3.1.

### Chromatin immunoprecipitation (ChIP)

ChIP was performed as described previously^72^. Briefly, cells were crosslinked with formaldehyde for 10 min at room temperature. Crosslinking was quenched by 2.5-M glycine and incubated for 5 min at room temperature. After formaldehyde crosslinking, cells were washed with PBS and nuclei isolated in a buffer containing 50-mM HEPES-KOH, pH 7.5, 140-mM NaCl, 1-mM EDTA, 10% glycerol, 0.5% NP40, 0.25% TritonX, and protease inhibitors. Nuclei were washed with a buffer containing 10-mM Tris-HCl, pH 8.0, 200-mM NaCl, 1-mM EDTA, 0.5-mM EGTA, and protease inhibitors. Lysis and immunoprecipitations were carried out in a buffer containing 10-mM Tris-HCl, pH 8.0, 100-mM NaCl, 9-mM EDTA, 0.5-mM EGTA, 0.1% Na-Deoxycholate, 0.5% N-lauryolsarcosine, and 1.1% Triton X-100. Complexes were washed five times in RIPA buffer (50-mM HEPES, 500-mM LiCl, 0.1-mM EDTA, 1.0% NP40, and 0.7% Na-Deoxycholate) and once with TE buffer containing 50-mM NaCl. To elute, beads were incubated with agitation at 65 °C for 30 min. Two hundred microliter eluate was removed to a fresh tube, and all samples were reverse-crosslinked overnight at 65 °C for a minimum of 12 h, but not more than 18 h. Two hundred microliter 1xTE was added to reverse-crosslinked DNA to dilute SDS, and samples were RNaseA-treated (final 0.2-mg/mL RNase; 37 °C for 2 h) and Proteinase K (final 0.2-mg/mL Proteinase K; 55 °C for 2 h) before phenol:chloroform extraction and resuspension in 10-mM Tris-HCl pH 8.0. For ChIP–Western blot, the samples were reverse-crosslinked overnight at 65 °C and about 35–50 µg of protein sample was loaded on 4–20% Mini-protean TGX gradient gel (BioRad). The enrichment of SOX4 and SMARCA4 on TGFBR2 promoter and enhancer was calculated by ChIP-qPCR using gene-specific primers, normalized to the negative control region and presented relative to either non-specific IgG control or no dox treatment. The PNOC promoter was selected as negative control region as it has been previously noted to be predominantly expressed in tissue from the central nervous system but is not expressed in human breast tissues^73^. Primers used are human TGFBR2 promoter—Forward: 5′- TATCATGGCAAAGACCACCA**-**3′, Reverse: 5′- CCTACATTTGCCCAAGTTCC-3′; human TGFBR2 enhancer—Forward: 5′- GGGCTATGGCTCTACTGGAA-3′, Reverse: 5′- AAGGCTGTACCCCTGGAAAG-3′ and human PNOC promoter—Forward: 5′- GCTTGAGCTCCTTGGATGAC -3′, Reverse: 5′- CCTGTCCCTTACTGCAGA-3′.

### Analyses of ChIP-seq data

SMARCA4 (GSE72141), H3K27ac and H3K4me3 (GSE85158) ChIP-Seq raw data from MDA-MB-231 cells^51,52^ were acquired from GEO. The data were reanalyzed and the FASTQ files were aligned using BowTie2 (v2.2.6)^74^ to UCSC hg19 genome. Significantly enriched peaks were called using MACS (v1.4.2 20120305)^75^. Genes associated with significantly enriched peaks were determined using GREAT (v3.0.0) to identify genes within 10 kb of the summit centered peaks^76^ and visualized using IGV^77^.

### Chromatin accessibility assay

Chromatin accessibility assays were performed as described previously^48,78^ with minor modifications. Approximately 1.0 × 10^6^ HCC1143 or HCC1954 cells were subcultured for siRNA-mediated knockdown of SOX4 for 96 h. Following knockdown, nuclei were collected in nucleus preparation buffer (300-mM sucrose, 10-mM Tris-HCl at pH 7.5, 15-mM NaCl, 60-mM KCl, 5-mM MgCl2, 0.1-mM EDTA, 0.15-mM spermine, 0.5-mM spermidine, 0.1% Nonidet-P40, 0.5-μM phenylmethyl sulfonyl fluoride) supplemented with 3-mM CaCl2, pelleted by centrifugation, resuspended in MNase digestion buffer (nucleus preparation buffer without Nonidet-P40 and supplemented with 3-mM CaCl2) ± 5 U of MNase (Sigma, N3755), and incubated for 10 min at 37 °C. Reactions were terminated with 20-μl stop solution (100-mM EDTA, 10-mM EGTA [pH 8.1]) and 10-μl SDS 10% (w/v). Chromatin was incubated for 5 h at 65 °C with proteinase K to reverse cross-link and remove protein followed by incubation with RNaseA to remove RNA. DNA was extracted with phenol–chloroform–isoamyl alcohol extraction followed by isopropanol precipitation and resuspended in 10-mM Tris [pH 8.0]. DNA recovered from “Cut” (+MNase) and “Uncut” (-MNase) samples were used in qRT-PCR assays to measure the relative abundance of targeted regions using gene-specific primer pairs. Chromatin accessibility ratios were calculated by determining the “Uncut” to “Cut” ratio for independent pairs of samples, normalized to the negative control region, PNOC promoter, and presented relative to either non-specific IgG control or no dox treatment.

### Statistical analysis

Data are represented as mean and standard deviation of at least three independent experiments. Statistical analyses were carried out with Graphpad Prism version 7.0 and differences were analyzed by unpaired two-tailed *t*-test between two groups and by two-way ANOVA followed by Tukey pairwise comparison test for differences between more than two groups.

## Supplementary information

Supplementary Information

Supplementary Data 1

Supplementary Data 2

Supplementary Data 3

Supplementary Data 4

Supplementary Data 5

## Data Availability

The data generated and analysed during this study are described in the following data record: 10.6084/m9.figshare.14141474^79^. RNAseq data have been deposited in the Gene Expression Omnibus (GEO) under accession number https://identifiers.org/geo:GSE158295^80^. Proteomic data from MIB/MS (https://identifiers.org/pride.project:PXD022596^81^) and IP-MS (https://identifiers.org/pride.project:PXD022811^82^) analyses have been made available through the PRIDE database. SMARCA4 (https://identifiers.org/geo:GSE72141^83^), H3K27ac and H3K4me3 (https://identifiers.org/geo:GSE85158^84^) ChIP-Seq raw data from MDA-MB-231 cells were acquired from Gene Expression Omnibus (GEO). The data for Figs. 1, 2, and 5 are presented in Supplementary Data 1–5, which are also available in Excel format as part of the figshare data record.
