# Supplementary material for: SOX4 and SMARCA4 cooperatively regulate PI3k signaling through transcriptional activation of TGFBR2
Source: NPJ Breast Cancer. 2021 Apr 9;7:40. doi: 10.1038/s41523-021-00248-2 (PMC8035213; doi:10.1038/s41523-021-00248-2)

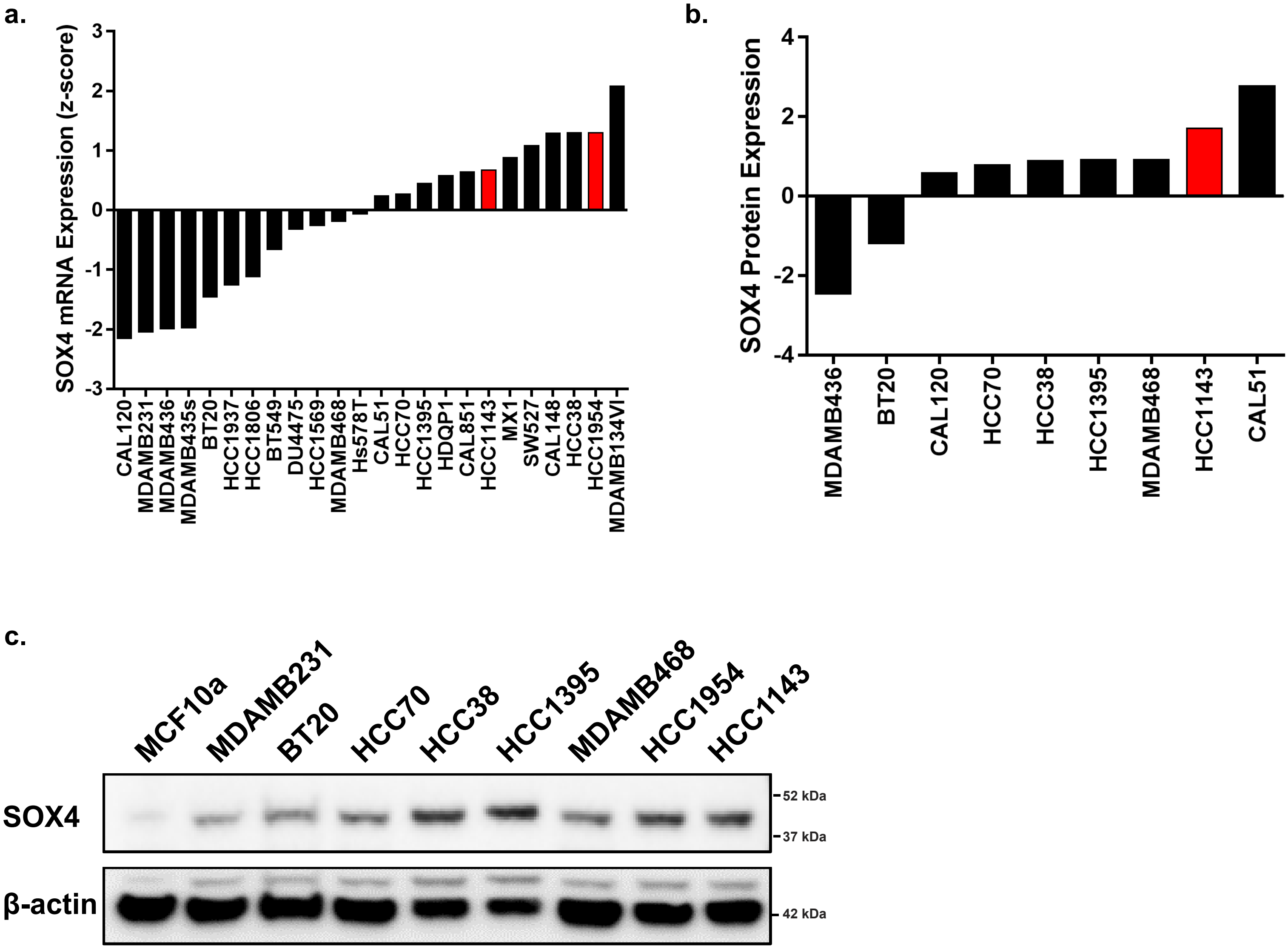

Supplementary Figure 1. Identification of basal-like breast cancer cell lines with high SOX4 expression. Analysis of the SOX4 mRNA (a) and protein (b) expression in a panel of basal-like breast cancer cell lines from the CCLE dataset.(c) Immunoblot analysis validating the expression of SOX4 in basal-like breast cancer cell lines selected from the CCLE panel based on high and low SOX4 expression.

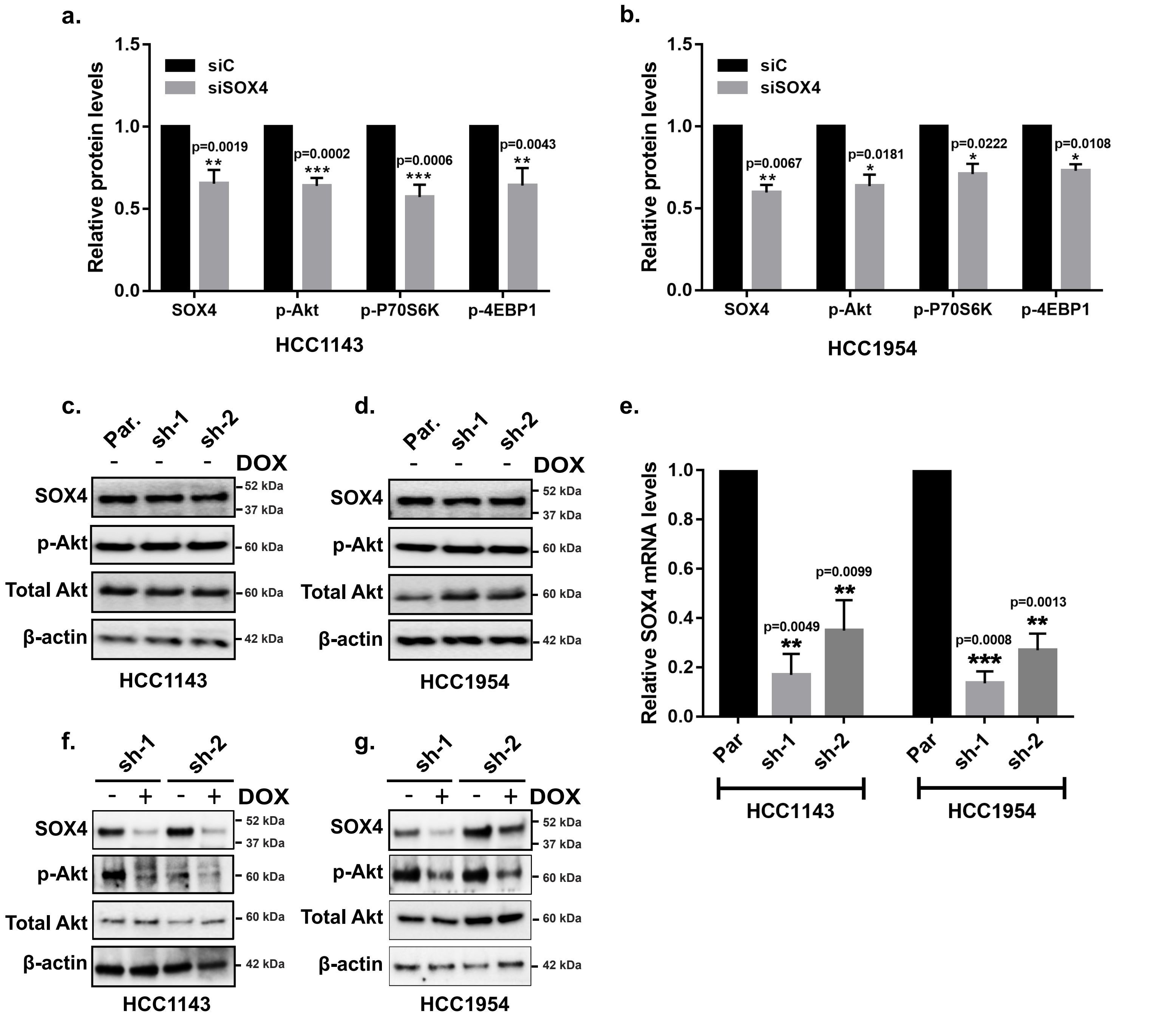

Figure S2. SOX4 mediates activation of PI3K/Akt signaling. Western blot quantification from three independent experiments using Image J software demonstrating significant reduction in protein levels of SOX4, p-Akt, p-P70S6K and p-4EBP1 following siRNA mediated knockdown of SOX4 compared to control siRNA in HCC1143 (a) and HCC1954 (b) cell lines respectively. HCC1143 and HCC1954 cells were engineered to express one of two tetracycline inducible shRNA against SOX4 (sh-1 and sh-2). Basal SOX4, p-Akt and total Akt protein levels are consistent in parental and sh1 or sh2 expressing HCC1143 (c) or HCC1954 (d) cell lines (e) Doxycycline treatment down regulates SOX4 expression in HCC1143 (2 μg/ml dox) (sh-1=83.0%, p=0.005; sh-2=65.0%, p=0.01) as well as in HCC1954 (1 μg/ml dox) (sh- 1=86.5%, p=0.0008; sh-2=73.0%, p=0.001) cells compared to their respective parental untreated controls. Immunoblots showing reduced SOX4 expression and Akt phosphorylation at S473 in HCC1143 (f) and HCC1954 (g) cell lines by both shRNA's when treated with doxycycline.

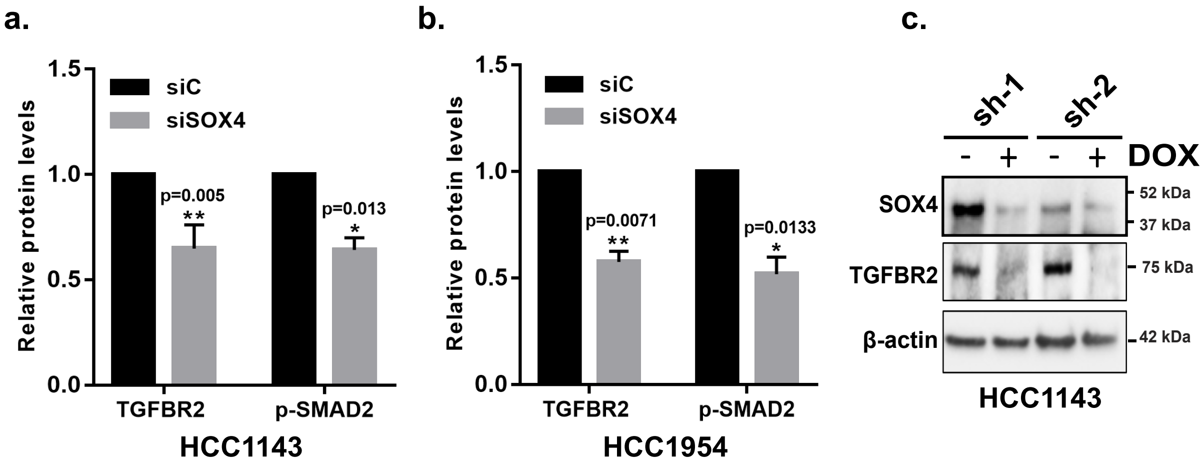

Supplementary Figure 3. SOX4 regulates TGFBR2 expression. Western blot quantification from three independent experiments using Image J software demonstrating significant reduction in protein levels of TGFBR2 following siRNA mediated knockdown of SOX4 compared to control siRNA in HCC1143 (a) and HCC1954 (b) cell lines respectively. Western blots showing reduced SOX4 and TGFBR2 expression in HCC1143 (b) and HCC1954 (c) cell lines by shRNA (sh-1 and sh-2) when treated with doxycycline.

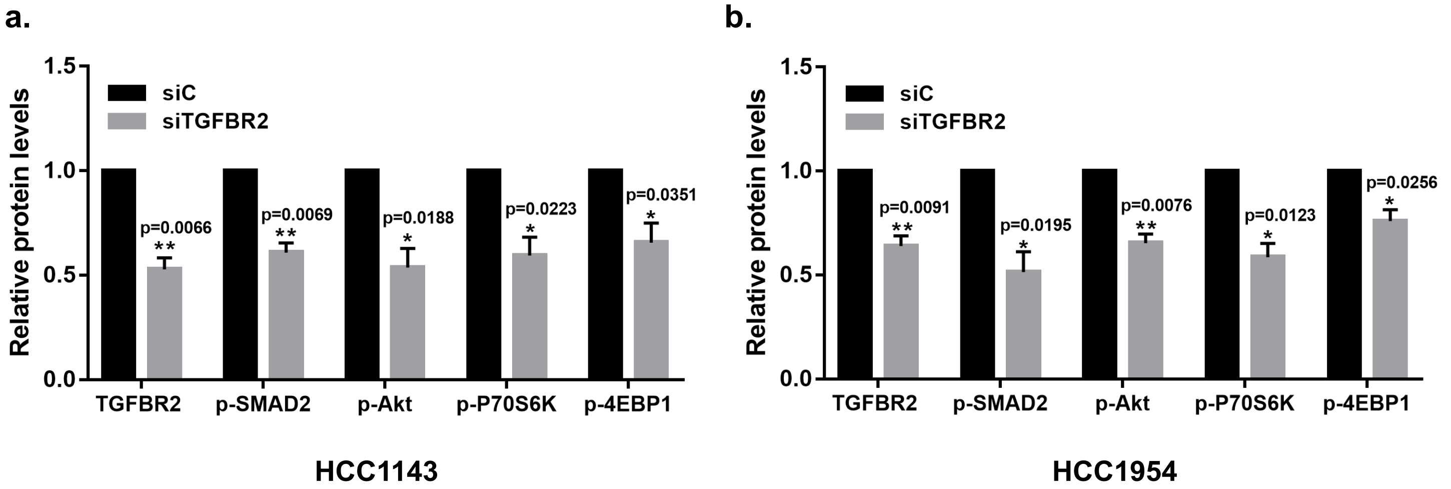

Supplementary Figure 4. TGFBR2 regulates PI3K/Akt pathway activity in basal-like breast cancer. Western blot quantification from three independent experiments using Image J software demonstrating significant reduction in protein levels of TGFBR2, p-SMAD2, p-Akt, p-P70S6K and p-4EBP1 following siRNA mediated knockdown of TGFBR2 compared to control siRNA in HCC1143 (a) and HCC1954 (b) cell lines respectively.

a.

b.

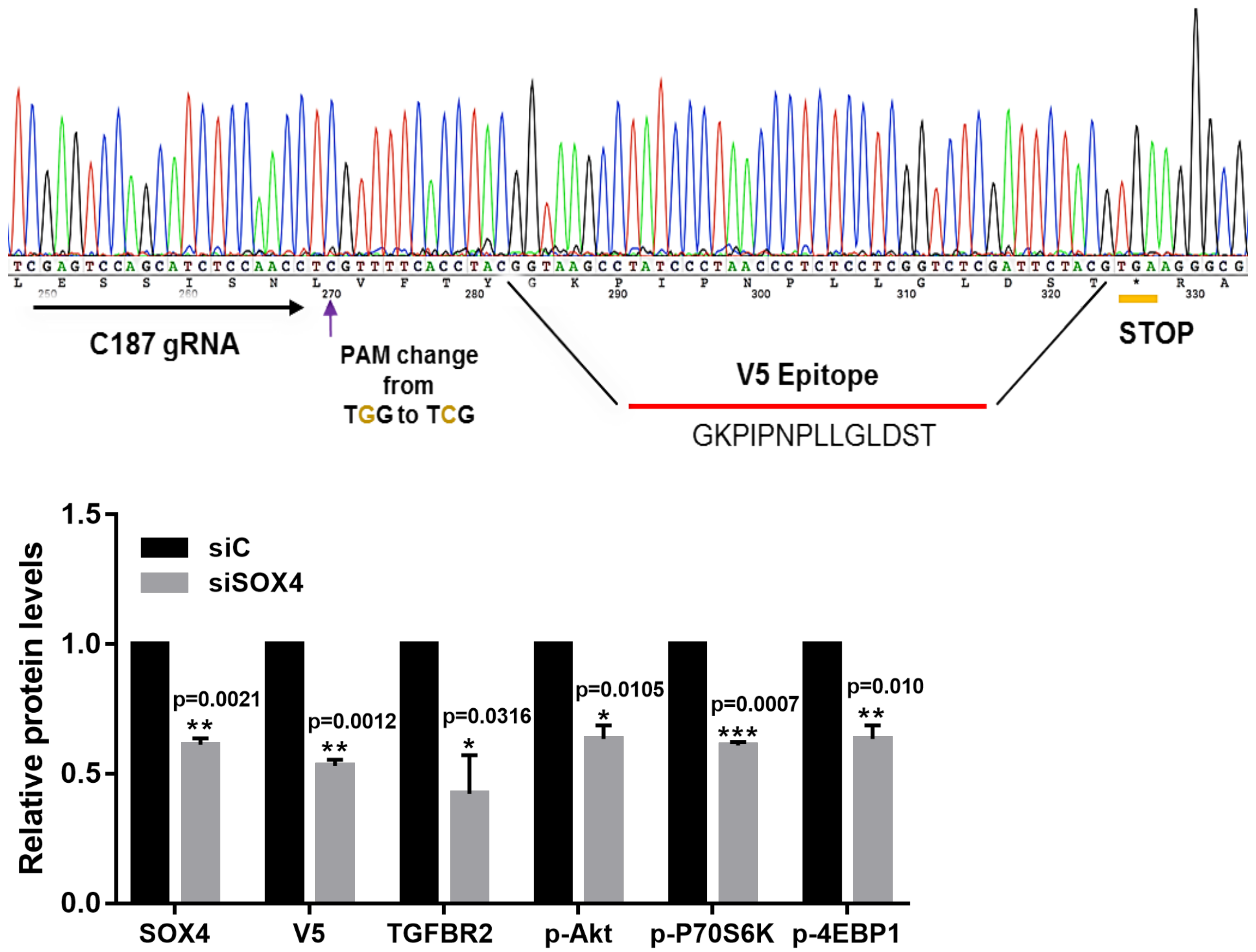

Supplementary Figure 5. Schematic representation of the V5 epitope tagging in the SOX4 3' locus. (a) C187 gRNA used for targeting of Cas protein is marked with bold arrow. PAM motif at residue 270 was altered from G to C. V5 epitope sequence used to tag the SOX4 3' locus is highlighted with red line and the stop codon TGA is marked with yellow solid bar. Western blot quantification from three independent experiments using Image J software demonstrating significant reduction in protein levels of SOX4, V5, TGFBR2, p-SMAD2, p-Akt, p-P70S6K and p-4EBP1 following siRNA mediated knockdown of TGFBR2 compared to control siRNA in HCC1143 (b) and HCC1954 (c) cell lines respectively.

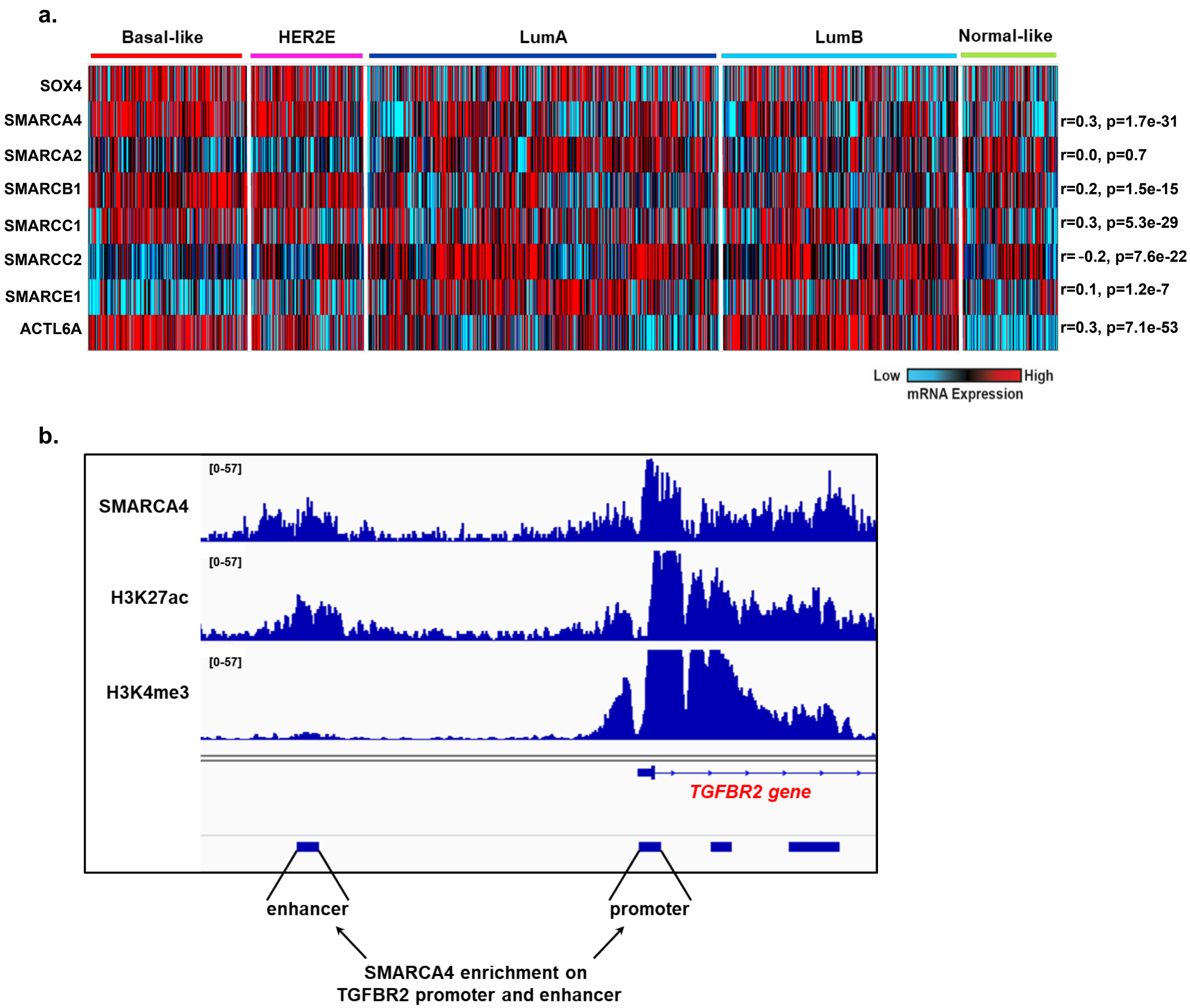

Supplementary Figure 6. SWI/SNF core subunits are up-regulated in basal-like tumors from METABRIC dataset and interact with SOX4. (a) mRNA expression patterns of SWI/SNF complex core subunit genes relative to SOX4 were determined for 1,986 human breast tumor samples from the METABRIC dataset. Samples are organized by PAM50 molecular subtype; red indicates high mRNA expression, and blue depicts low mRNA levels. (b) Visualization of the SMARCA4, H3K27ac and H3K4me3 enrichment profile on TGFBR2 promoter and enhancer regions obtained from the analysis of publicly available ChIP-seq dataset in MDA-MB-231 cell line (GSE72141 and GSE85158).

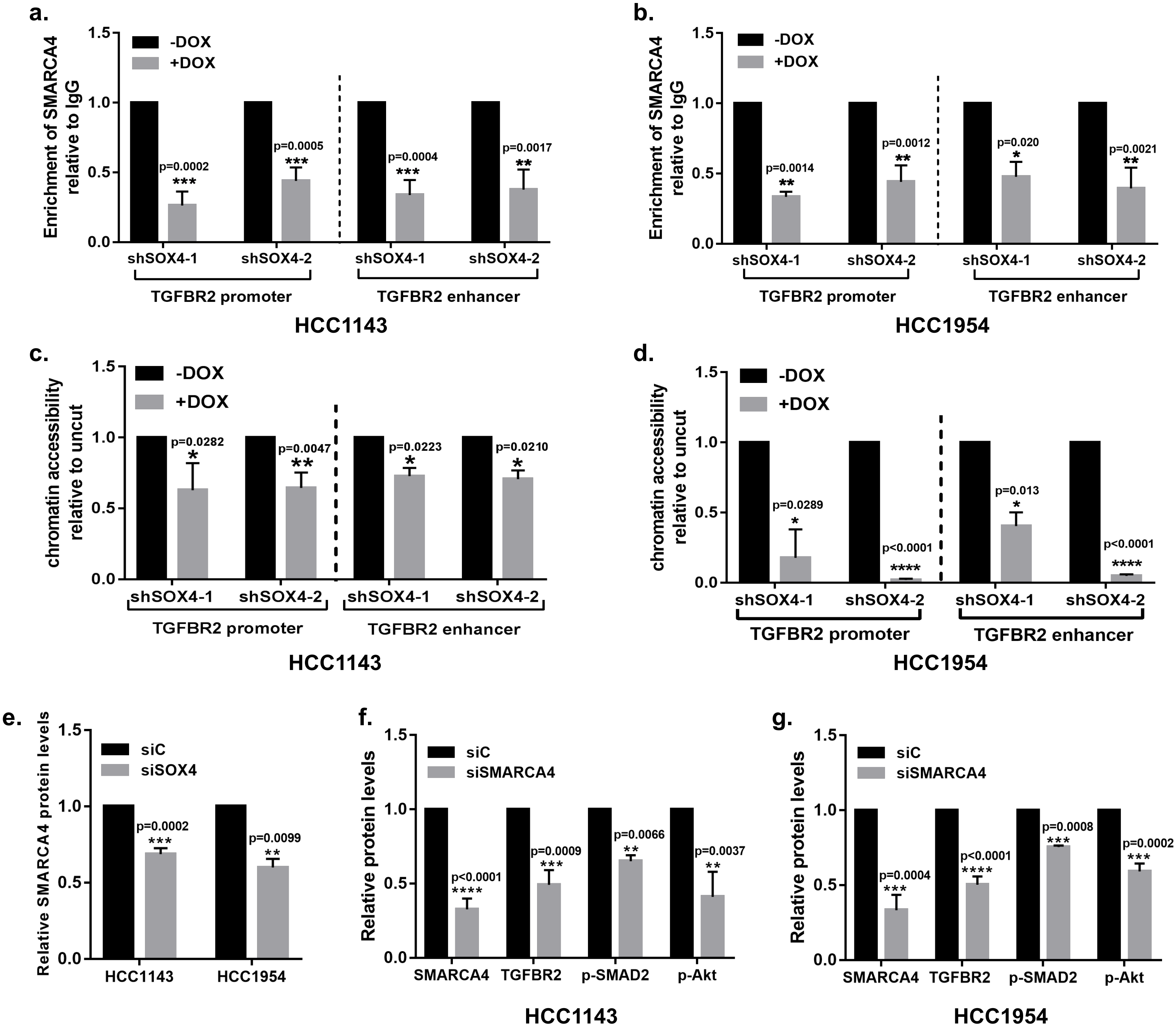

Supplementary Figure 7. SOX4 promotes SMARCA4 recruitment to mediate open chromatin conformation at TGFBR2 regulatory regions. ChIP-qPCR demonstrating reduction in SMARCA4 enrichment when treated with doxycycline in HCC1143 at both TGFBR2 promoter (sh-1=73.6%, p=0.0002; sh-2=56.1%, p=0.0006) and TGFBR2 enhancer (sh-1=66.2%, p=0.0004; sh-2=62.3%, p=0.002) regions. (b) Similar reduction in enrichment was observed in HCC1954 cells upon doxycycline treatment at both TGFBR2 promoter (sh-1=66.5%, p=0.001; sh-2=56.0%, p=0.001) and TGFBR2 enhancer (sh-1=52.3%, p=0.02; sh-2=60.6%, p=0.002) regions. (c) Chromatin accessibility assay demonstrating that accessibility was reduced upon doxycycline treatment in HCC1143 at both TGFBR2 promoter (sh-1=37.1%, p=0.03; sh- 2=35.6%, p=0.004) and TGFBR2 enhancer (sh-1=27.2%, p=0.02; sh-2=29.3%, p=0.02) regions. (d) Similar reduction in accessibility was observed in HCC1954 cells upon doxycycline treatment at both TGFBR2 promoter (sh-1=82.1%, p=0.03; sh-2=99.8%, p<0.0001) and TGFBR2 enhancer (sh-1=59.6%, p=0.01; sh-2=95.1%, p<0.0001) regions. (e) Western blot quantification from three independent experiments using Image J software demonstrating significant reduction in protein levels of SMARCA4 following siRNA mediated knockdown of SOX4 compared to control siRNA in HCC1143 and HCC1954 cell lines. Immunoblot quantification from three independent experiments using Image J software demonstrating significant reduction in protein levels of SMARCA4, TGFBR2, p-SMAD2 and p-Akt, following siRNA mediated knockdown of SMARCA4 compared to control siRNA in HCC1143 (f) and HCC1954 (g) cell lines respectively.

Supplementary Dataset information:	
Supplementary Data 1	TCGA tumor sample summary
Supplementary Data 2	METABRIC tumor sample summary
Supplementary Data 3	Summary of normalized LFQ intenstities for significant kinases after SOX4 KD in HCC1143 cell line
Supplementary Data 4	Summary of TPM values for the significantly altered kinases from MIB/MS analysis
Supplementary Data 5	Potential SOX4-interacting protein candidates identified by LC-MS/MS

Supplementary Figure 8. Raw data for the blots included in each figure and supplementary data.

For Figure 1e.

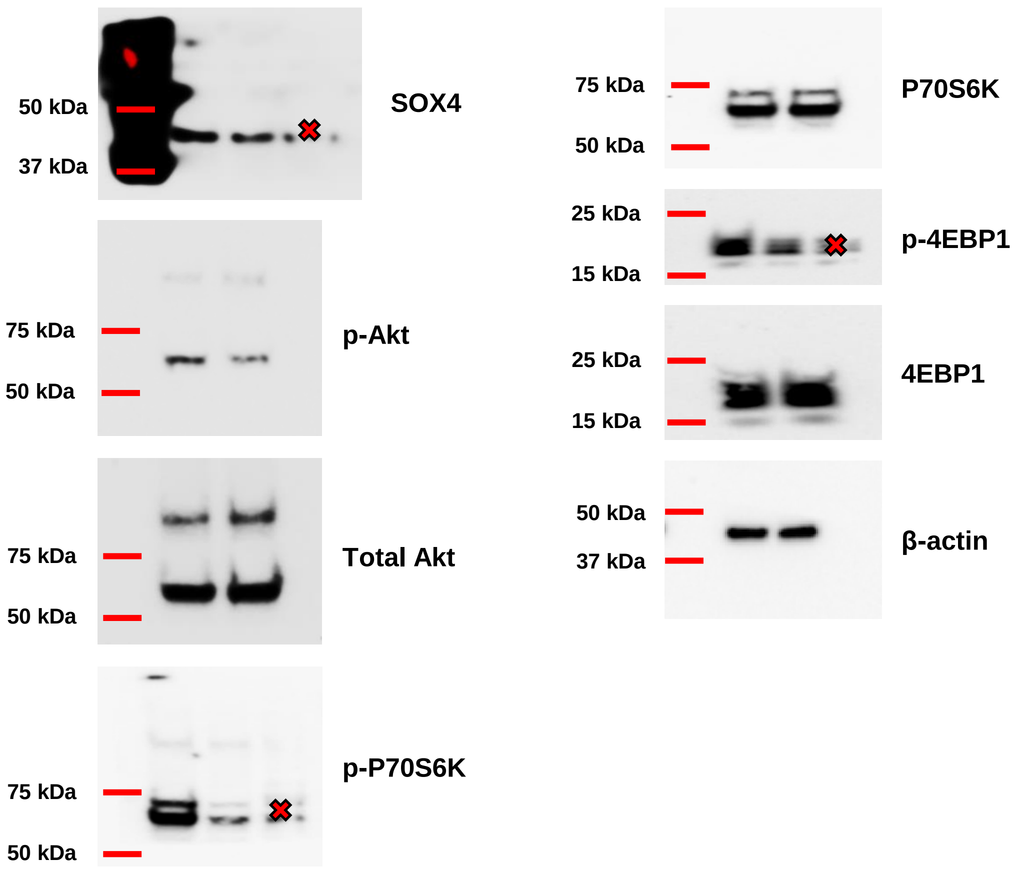

For Figure 1f.

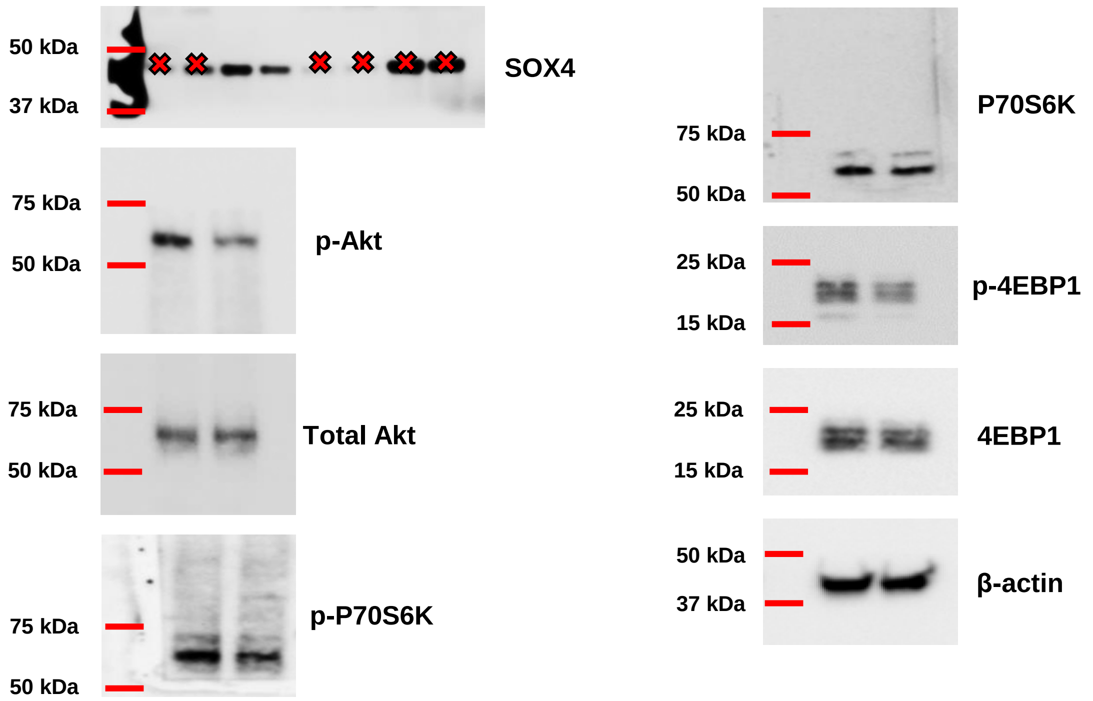

Supplementary Figure 8. Raw data for the blots included in each figure and supplementary data.

For Figure 2e.

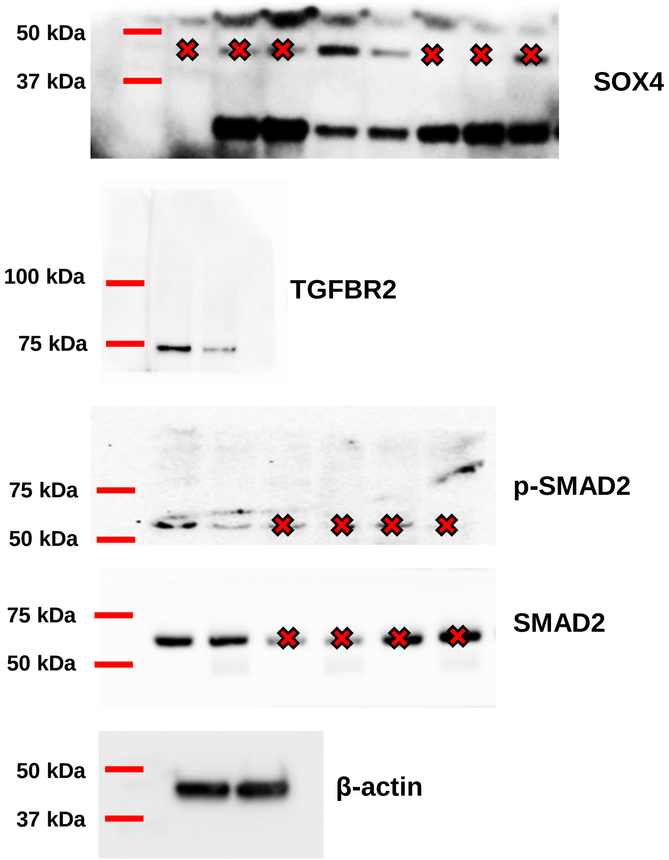

For Figure 2f.

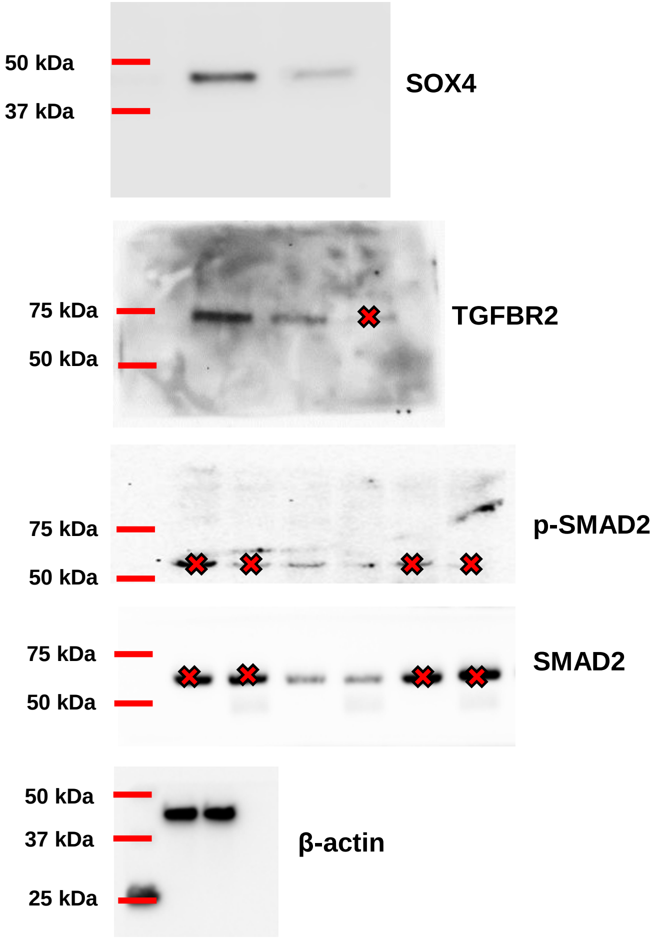

Supplementary Figure 8. Raw data for the blots included in each figure and supplementary data.

For Figure 3a.

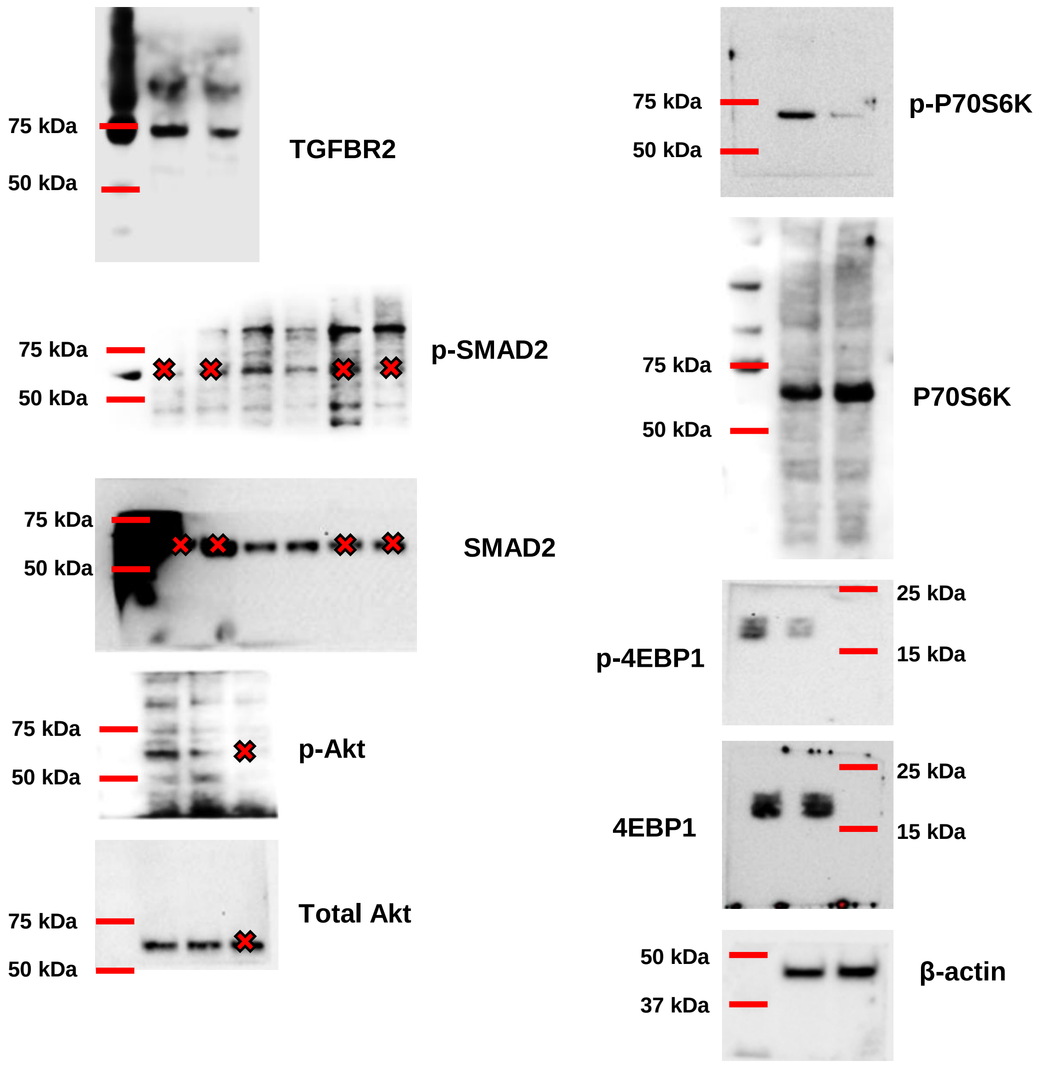

For Figure 3b.

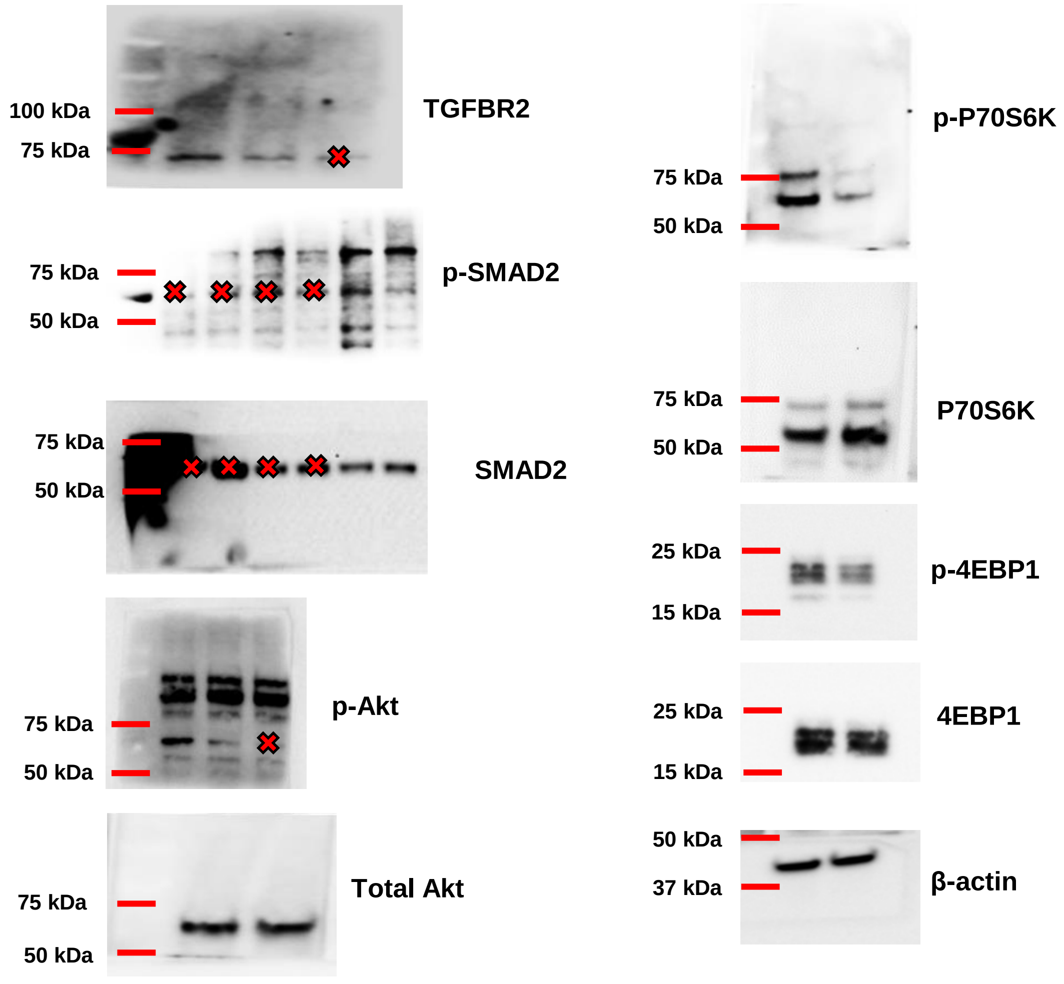

Supplementary Figure 8. Raw data for the blots included in each figure and supplementary data.

For Figure 3c.

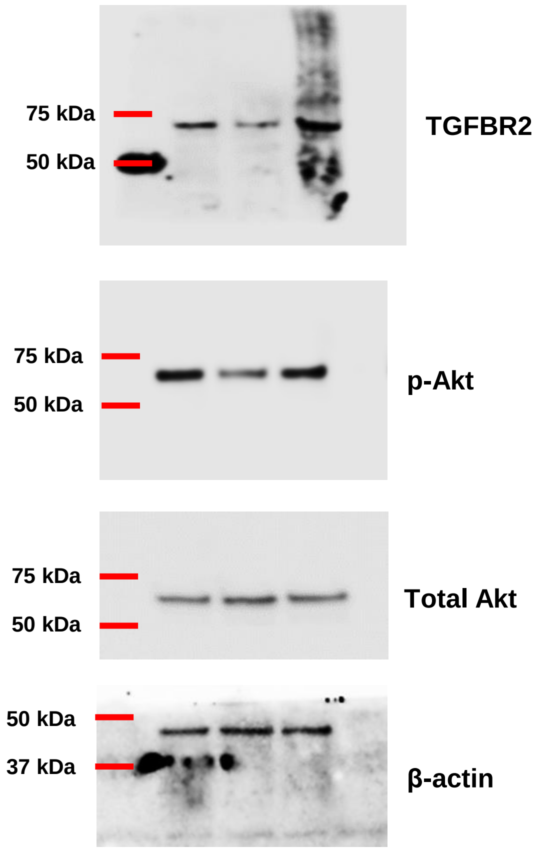

For Figure 3d.

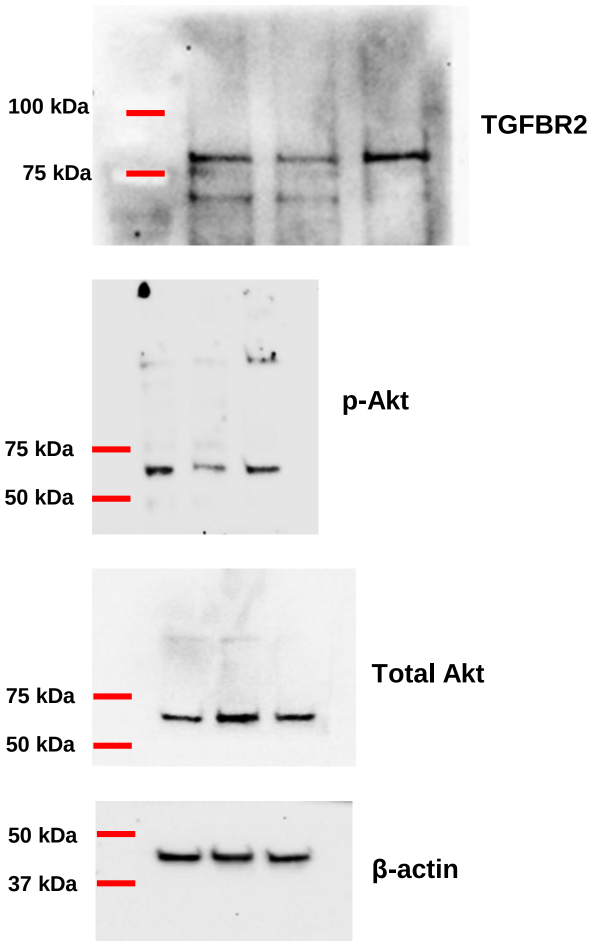

Supplementary Figure 8. Raw data for the blots included in each figure and supplementary data.

For Figure 4c.

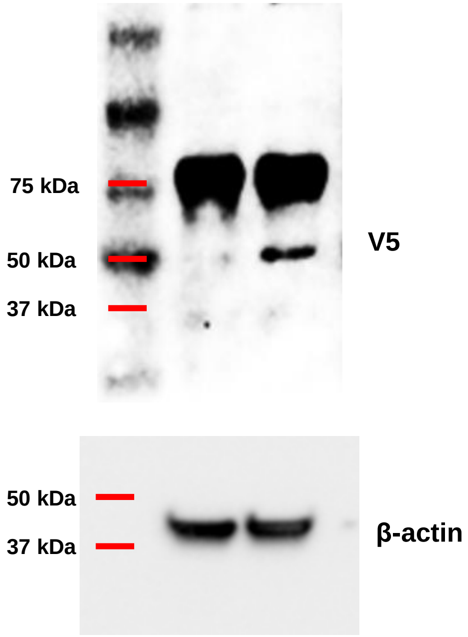

For Figure 4f.

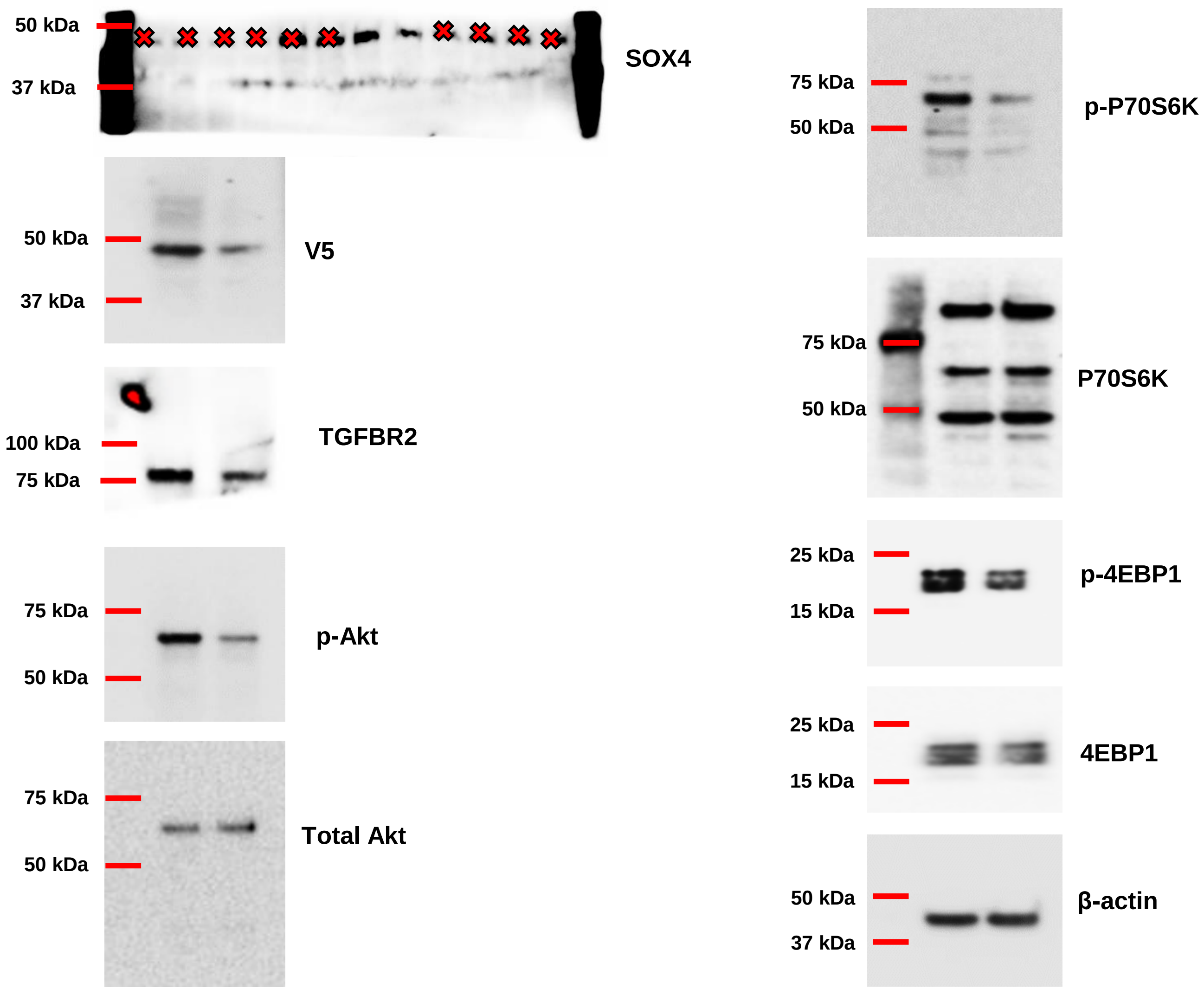

Supplementary Figure 8. Raw data for the blots included in each figure and supplementary data.

For Figure 5c.

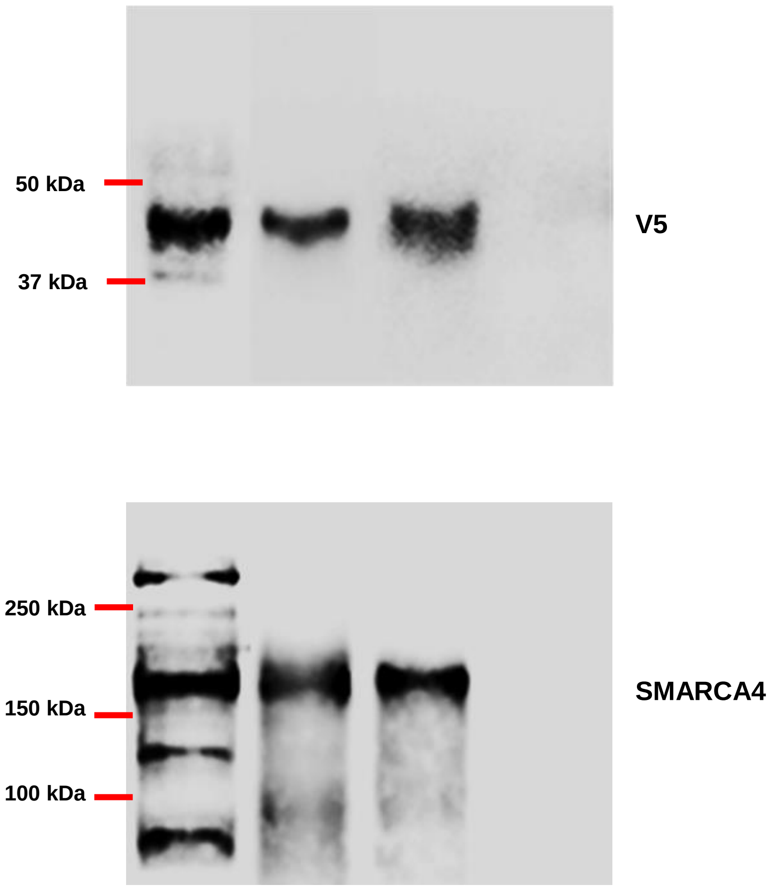

For Figure 5d.

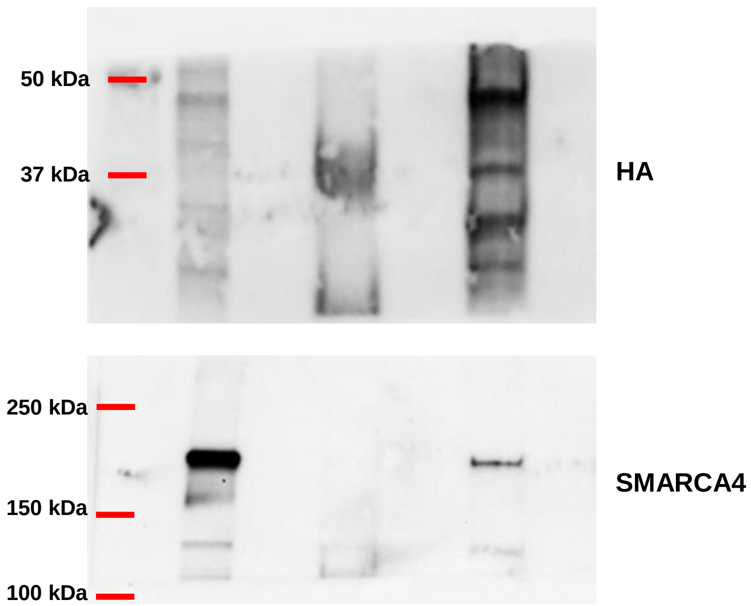

Supplementary Figure 8. Raw data for the blots included in each figure and supplementary data.

For Figure 6c.

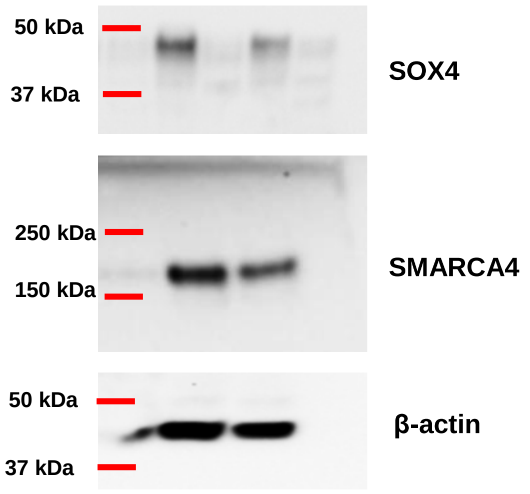

For Figure 6e.

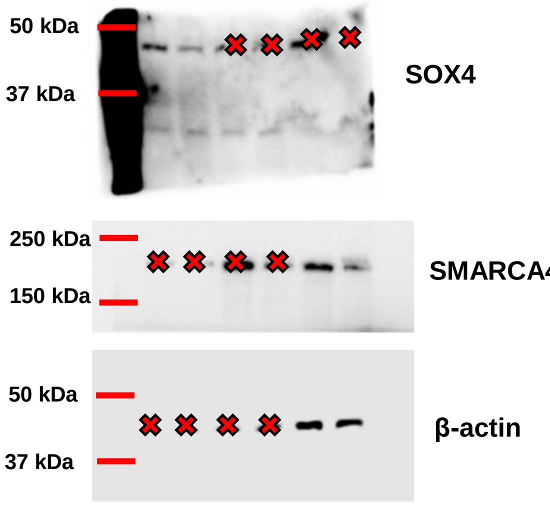

For Figure 6d.

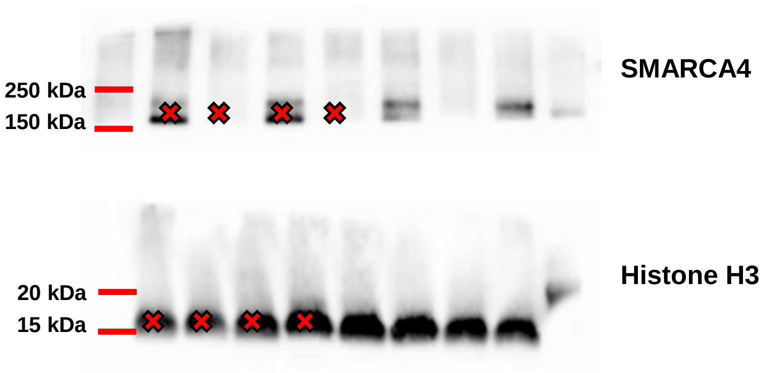

For Figure 6f.

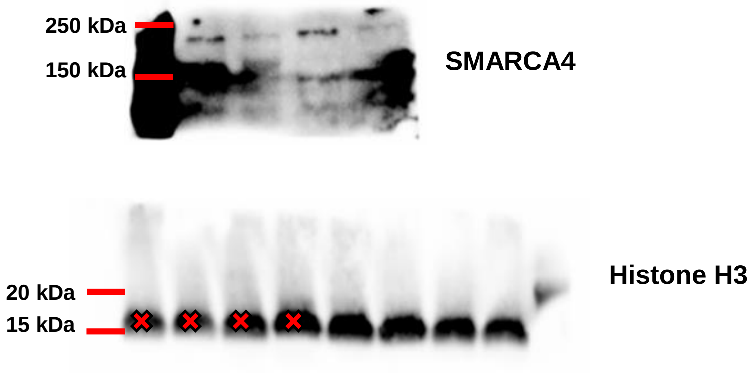

Supplementary Figure 8. Raw data for the blots included in each figure and supplementary data.

For Figure 6k.

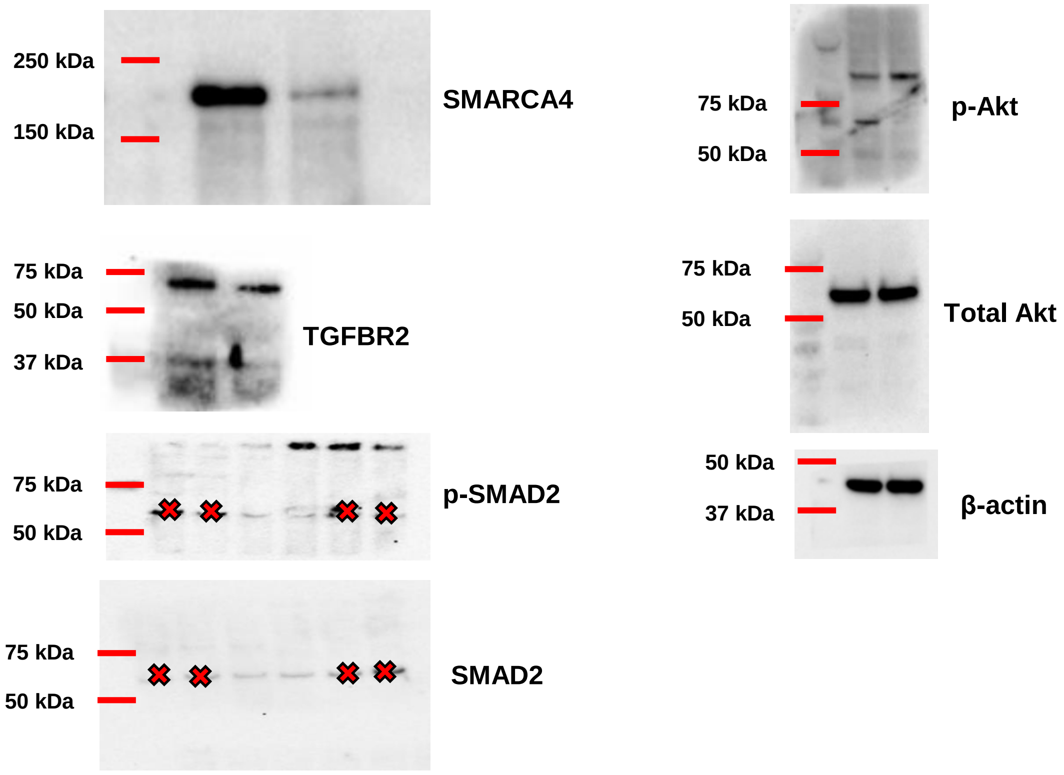

For Figure 6l.

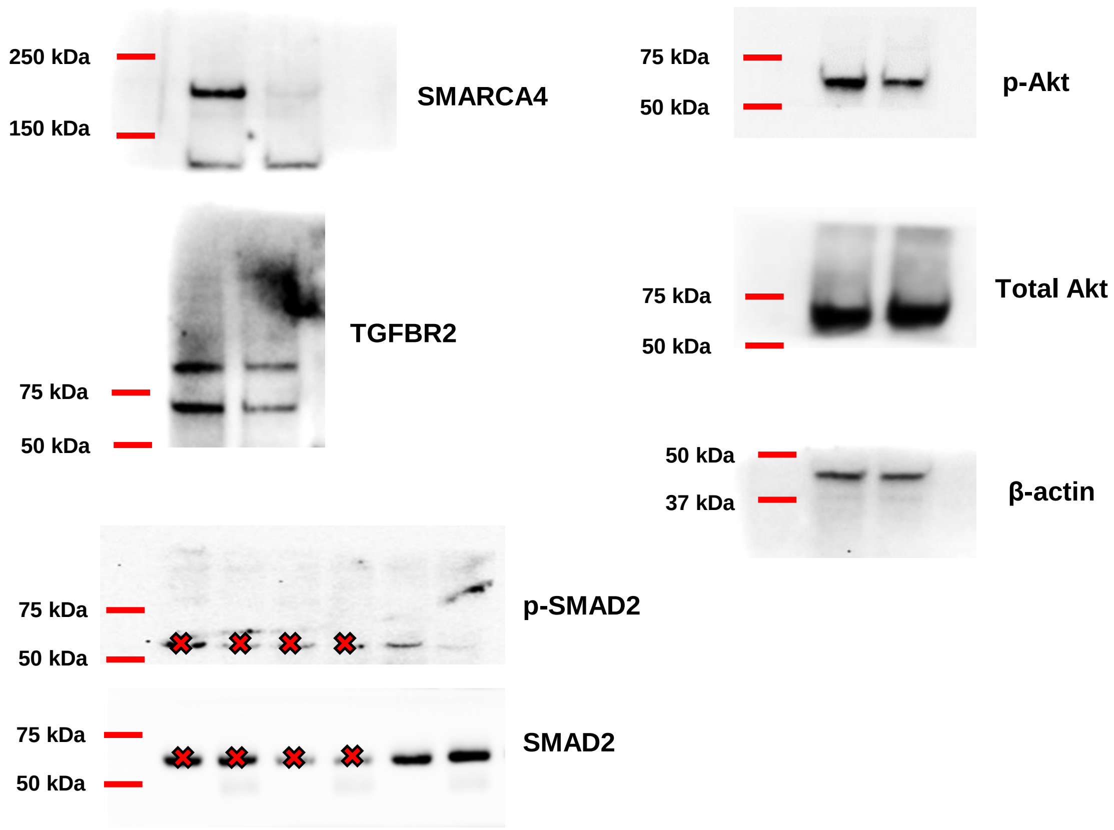

Supplementary Figure 8. Raw data for the blots included in each figure and supplementary data.

For Supplementary Figure 1c.

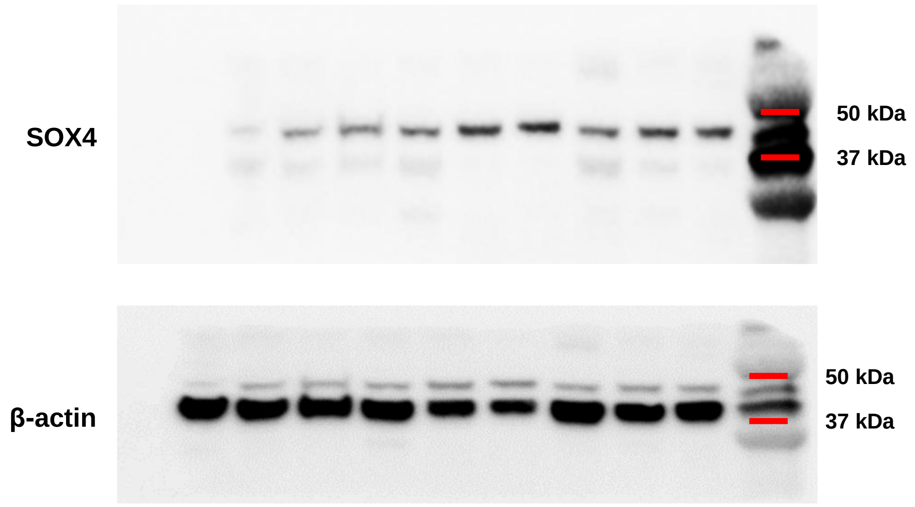

Supplementary Figure 8. Raw data for the blots included in each figure and supplementary data.

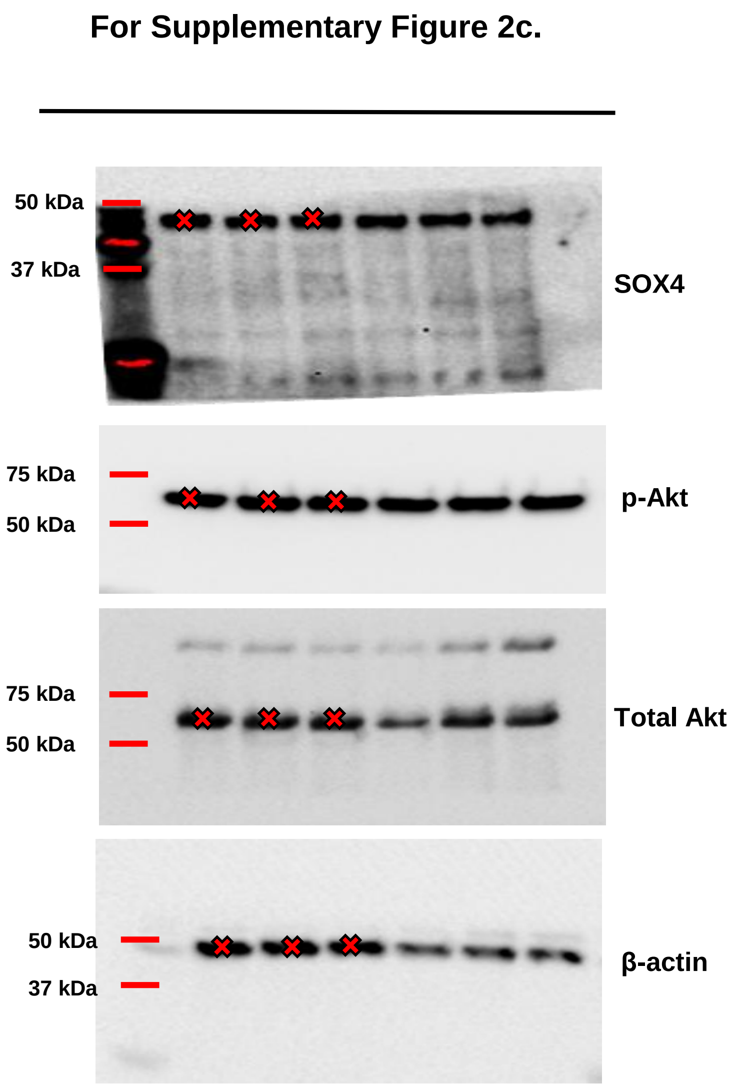

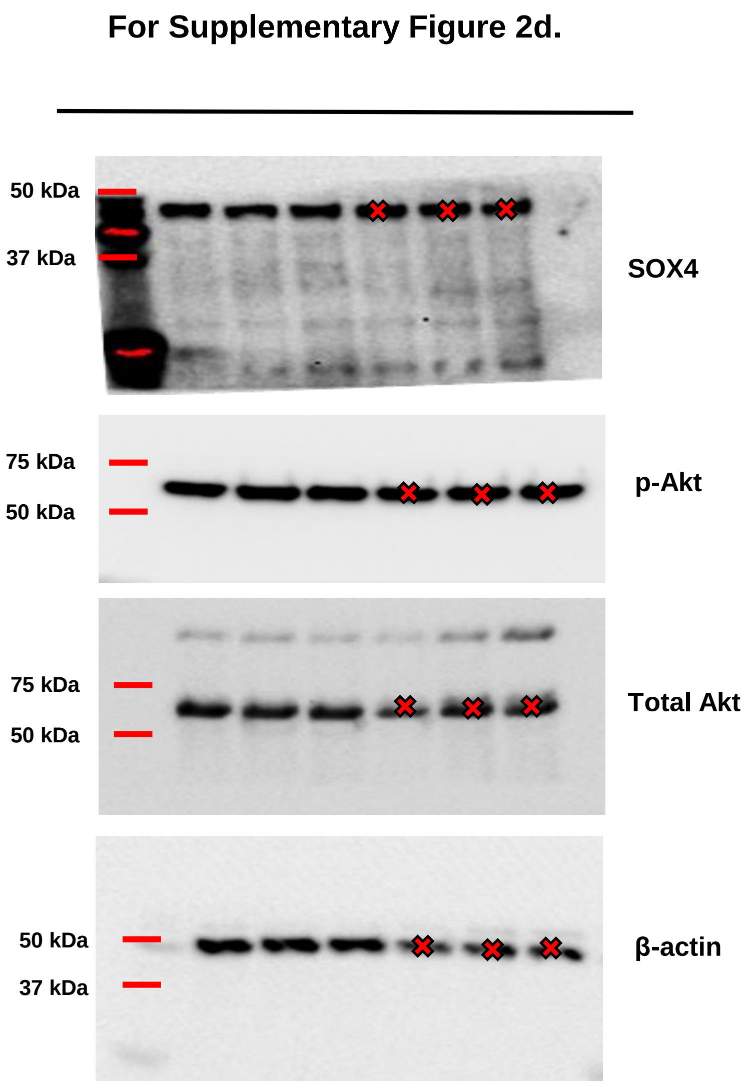

Supplementary Figure 8. Raw data for the blots included in each figure and supplementary data.

For Supplementary Figure 2f.

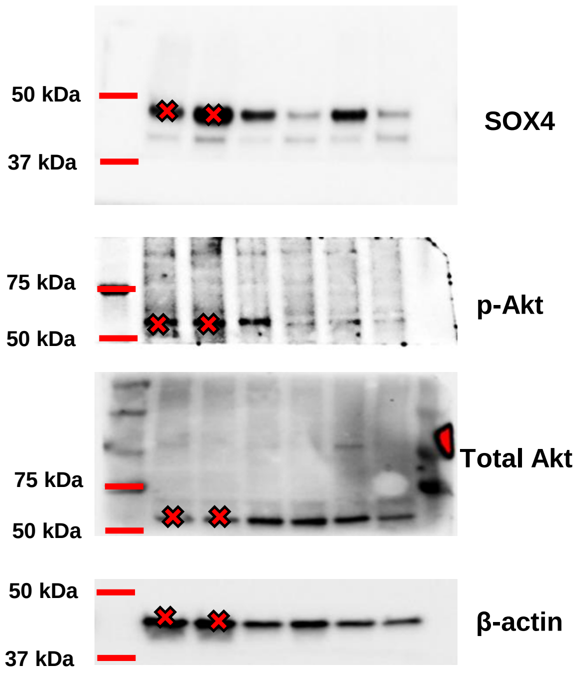

For Supplementary Figure 2g.

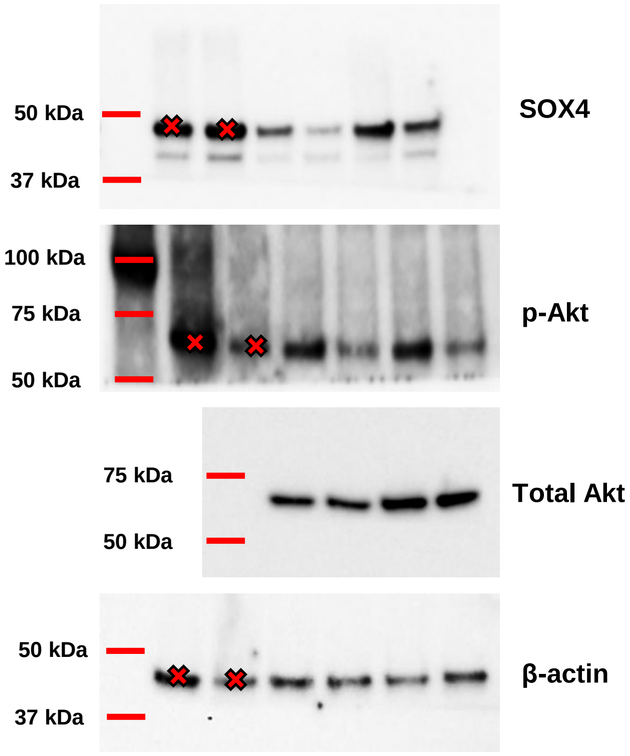

Supplementary Figure 8. Raw data for the blots included in each figure and supplementary data.

For Supplementary Figure 3c.

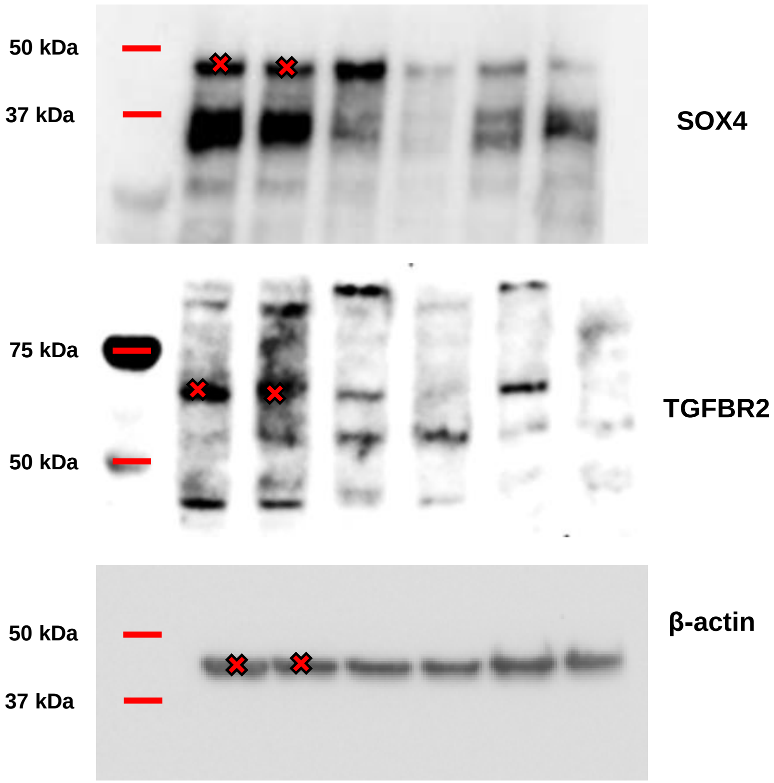

For Supplementary Figure 3d.

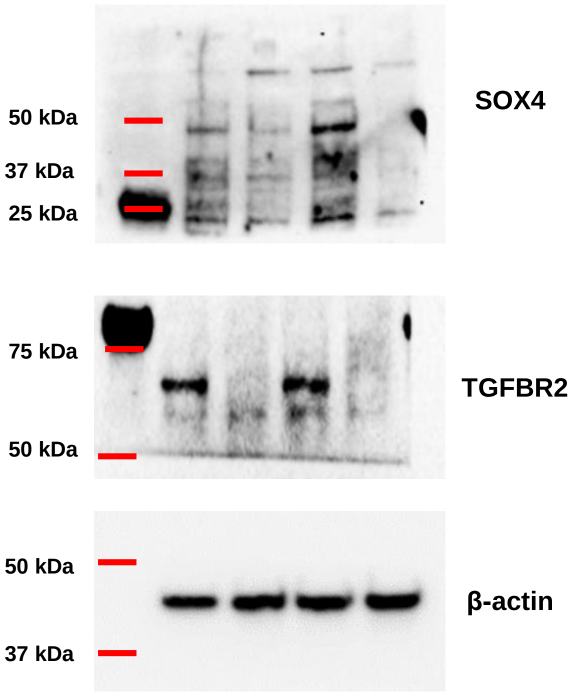

Supplement: Supplementary file 1 — Supplementary Information [file 41523_2021_248_MOESM1_ESM.pdf]
